# Promoter Hypermethylation‐Induced Silencing of FXYD1 Drives Breast Cancer Metastasis via DDX5‐Mediated Wnt/β‐Catenin Pathway Activation

**DOI:** 10.1002/advs.202521921

**Published:** 2026-03-04

**Authors:** Ping Wen, Fanli Qu, Long Wang, Qing Shao, Sisi Li, Yang Qin, Dongping Jiang, Senmiao Zhang, Jiangdong Sui, Guanwen Wang, Ningning Zhang, Xiaohua Zeng

**Affiliations:** ^1^ Chongqing University Cancer Hospital School of Medicine Chongqing University Chongqing China; ^2^ Department of Breast Cancer Center Chongqing University Cancer Hospital Chongqing China; ^3^ Radiation Oncology Center Chongqing University Cancer Hospital Chongqing China; ^4^ Chongqing Key Laboratory for Intelligent Oncology in Breast Cancer (iCQBC) Chongqing University Cancer Hospital Chongqing China

**Keywords:** breast cancer metastasis, DDX5, FXYD1, promoter hypermethylation, Wnt/β‐catenin signaling

## Abstract

Epigenetic silencing of tumor suppressor genes is a hallmark of cancer progression. The FXYD (FXYD domain‐containing ion transport regulator) family, classically known for ion transport regulation, has recently been implicated in oncogenesis; however, the role of its founding member, FXYD1, in breast cancer remains unclear. Here, FXYD1 is identified as a significantly downregulated gene in breast cancer tissues, and low FXYD1 expression is associated with unfavorable patient prognosis. Integrative transcriptomic, clinical, and epigenetic analyses revealed that promoter hypermethylation drives FXYD1 silencing. Functional restoration of FXYD1 suppressed cell proliferation, migration, and lung metastasis both in vitro and in vivo. Mechanistically, FXYD1 acts as a nuclear scaffold that recruits the E3 ubiquitin ligase MAEA to the RNA helicase DDX5, a coactivator of β‐catenin, promoting K63‐linked ubiquitination and proteasomal degradation of DDX5. This process reduces β‐catenin stability, impairs its nuclear translocation, and attenuates Wnt target gene expression. Collectively, our findings uncover a previously unrecognized FXYD1–MAEA–DDX5 axis that inhibits Wnt/β‐catenin signaling through a non‐canonical ubiquitin–proteasome pathway, establishing FXYD1 as a tumor suppressor and potential prognostic biomarker and therapeutic target in breast cancer.

## Graphical abstract

This study identifies FXYD1 as an epigenetically silenced tumor suppressor in breast cancer. DNA methylation turns off the gene FXYD1 in breast cancer, and low levels predict worse outcomes. Restoring FXYD1 limits breast cancer cells proliferation and metastasis. In the nucleus, FXYD1 recruits the E3 ligase MAEA to K63‐ubiquitinate DDX5 for proteasomal degradation, dampening Wnt/β‐catenin signaling that drives aggressive behavior.

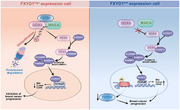

## 1 Introduction

Breast cancer remains the most commonly diagnosed malignancy and one of the leading causes of cancer‐related mortality among women worldwide [1]. Despite significant advances in early detection and the development of targeted therapies, tumor recurrence and metastasis, particularly in aggressive subtypes such as triple‐negative breast cancer (TNBC), continue to pose significant clinical challenges [2, 3, 4]. Mechanistically, breast cancer progression is highly heterogeneous, shaped by tumor‐intrinsic signaling rewiring and multilayer regulation, including epigenetic alterations, non‐coding RNAs, and dynamic tumor–microenvironment interactions across metastatic niches [5, 6]. Among these processes, accumulating evidence underscores epigenetic dysregulation as a central hallmark of breast cancer pathogenesis [7, 8, 9, 10]. In particular, epigenetic alterations frequently mediate the transcriptional silencing of tumor suppressor genes, disrupting key regulatory networks that maintain cellular homeostasis [11]. This silencing contributes not only to malignant transformation but also to sustained tumor growth, therapeutic resistance, and metastatic progression. Such epigenetic repression often occurs early in tumor development and may persist throughout disease evolution, underscoring its importance as both a mechanistic driver and a potential therapeutic target [11, 12, 13]. However, the full landscape of tumor suppressor genes subject to epigenetic inactivation in breast cancer, as well as the functional impact of their silencing, remains incompletely defined.

Disruption of ion transport and ionic homeostasis has been increasingly recognized as a driver of signaling rewiring and metastatic traits in cancer cells [14, 15]. The FXYD gene family encodes a group of small, single‐pass transmembrane proteins primarily known for their interaction with Na^+^/K^+^‐ATPase and their role in maintaining ion homeostasis [16]. Accumulating evidence links multiple FXYD members to cancer progression, although their effects appear highly context‐dependent across tumor types and molecular subtypes. For instance, FXYD5 has been most consistently associated with aggressive phenotypes, including invasion and metastasis, and with adverse outcomes in breast cancer and gastric cancer [17, 18]. FXYD3 has been implicated in tumor‐promoting phenotypes in several malignancies and was reported to support ER+ breast cancer stem‐like programs [19, 20, 21]. In addition, FXYD1 has been reported as an adverse prognostic factor and its enforced expression promotes tumor cell motility and invasiveness in ovarian cancer [22], whereas in colorectal cancer, TCGA‐based analyses indicate reduced FXYD1 expression with an inverse correlation between promoter methylation and FXYD1 mRNA levels, implicating epigenetic deregulation [23]. Importantly, recent reports further suggest that certain FXYD proteins may exert non‐canonical functions beyond ion transport, potentially influencing intracellular signaling, protein stability, and transcriptional regulation [24, 25]. Nevertheless, the biological significance and mechanistic contributions of the FXYD family in breast cancer remain largely undefined. Together, these observations motivated us to interrogate the FXYD family in breast cancer as a biologically related and experimentally tractable candidate set, and to determine whether specific members may exert previously unrecognized regulatory functions beyond their classical membrane‐associated roles.

In this study, we identify FXYD1 as a tumor suppressor that is specifically and consistently downregulated in breast cancer, primarily due to promoter hypermethylation. Notably, we demonstrate that FXYD1 is predominantly localized to the nucleus in breast cancer cells, where it exerts tumor‐suppressive effects by modulating oncogenic signaling networks. Mechanistically, we reveal that FXYD1 functions as a scaffold protein, facilitating the recruitment of the E3 ubiquitin ligase MAEA to DDX5, an RNA helicase and well‐established coactivator of β‐catenin. Specifically, DDX5 enhances β‐catenin–dependent transcription and may also stabilize β‐catenin by attenuating phosphorylation‐dependent degradation, thereby facilitating its accumulation and nuclear translocation [26, 27, 28]. By bridging MAEA and DDX5, FXYD1 enhances MAEA‐dependent K63‐linked polyubiquitination of DDX5 and its subsequent proteasomal degradation, a non‐canonical ubiquitin signaling mechanism that broadens the known functional scope of K63‐linked ubiquitination beyond its traditionally non‐degradative roles. Depletion of DDX5 results in reduced β‐catenin protein stability, impaired nuclear translocation, and suppressed transcription of downstream Wnt target genes. Consequently, Wnt/β‐catenin signaling is attenuated, leading to reduced tumor cell proliferation and metastatic potential.

Taken together, our findings reveal a previously unrecognized FXYD1–MAEA–DDX5 axis that regulates β‐catenin signaling via an unconventional ubiquitin‐proteasome pathway. This study not only uncovers a novel tumor‐suppressive function of FXYD1 in breast cancer but also highlights a broader paradigm in which scaffold proteins orchestrate proteostatic control of oncogenic effectors. These insights underscore the therapeutic potential of targeting the FXYD1–DDX5–β‐catenin signaling cascade as a strategy to counteract Wnt‐driven tumor progression in breast cancer.

## 2 Results

### 2.1 FXYD1 Is Downregulated in Breast Cancer and Serves as a Favorable Prognostic Factor

Given the established roles of FXYD family members in tumor progression, we first assessed the expression patterns and prognostic significance of all seven FXYD genes in breast cancer using public datasets (TCGA, KM‐Plotter, and METABRIC). Our analysis revealed that FXYD1, FXYD2, and FXYD6 were significantly downregulated, whereas FXYD3, FXYD4, and FXYD5 were upregulated in breast cancer tissues compared to normal tissues (Figure S1A). In the TCGA cohort, high expression of FXYD1, FXYD2, FXYD3, FXYD5, FXYD6, and FXYD7 correlated with favorable overall survival (OS), while FXYD4 emerged as the only unfavorable prognostic factor (Figure S1B–H). In the KM‐plotter dataset, higher expression of FXYD1, FXYD5, and FXYD6 was associated with improved OS (Figure S2A–G). In the METABRIC dataset, however, FXYD1 was the sole member consistently associated with favorable prognosis, whereas FXYD4, FXYD5, and FXYD6 were identified as unfavorable prognostic factors (Figure S3A–G). Although all FXYD proteins are classically recognized as modulators of Na^+^/K^+^‐ATPase transporter activity, the consistent downregulation of FXYD1 across datasets and its unique association with adverse clinical outcomes highlight its potential as a functionally significant tumor suppressor in breast cancer. Based on these observations, we focused our subsequent analyses on FXYD1.

**FIGURE 1:**
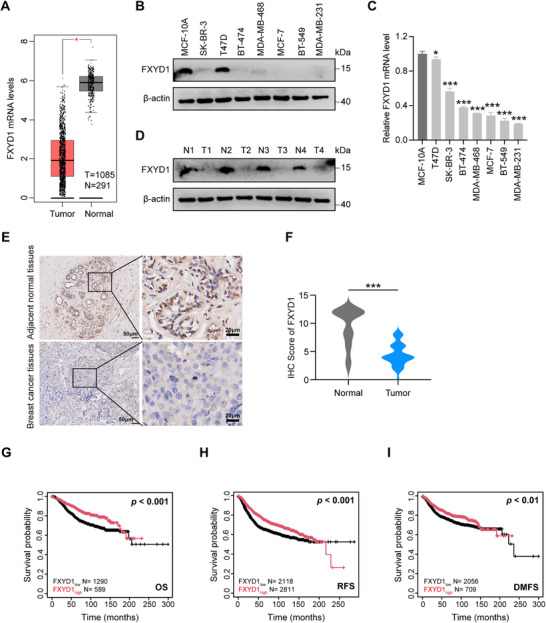
FXYD1 is downregulated in breast cancer and serves as a favorable prognostic factor. (A) GEPIA‐based analysis of FXYD1 expression in breast cancer tissues compared with normal tissues. (B, C) FXYD1 expression in breast cancer cell lines detected by immunoblotting (B) and qRT‐PCR (C). One‐way ANOVA. (D) Immunoblot analysis of FXYD1 expression in four paired breast cancer tissues (T, tumor; N, normal). (E, F) Representative images (E) and quantification of (F) of FXYD1 IHC staining in paired breast cancer tissues and normal breast tissues (n = 60 samples). one‐sided paired t‐test. (G–I) Kaplan–Meier survival analysis of OS (G, n = 1879), RFS (H, n = 4929), and DMFS (I, n = 2765) in breast cancer patients, from the KM Plotter database, stratified by FXYD1 expression using the optimal cut‐off value. Data are presented as the mean ± SD (n = 3 independent experiments). *p < 0.05, ***p < 0.001.

We next performed a multi‐platform evaluation to validate the clinical relevance of FXYD1 in breast cancer. Analysis of TCGA cohort via GEPIA consistently confirmed significantly lower FXYD1 expression in breast cancer tissues compared to normal tissues (Figure 1A). This result was further supported by immunoblotting and qRT‐PCR (Quantitative real‐time PCR) analyses, which showed marked downregulation of FXYD1 in a panel of seven breast cancer cell lines (MDA‐MB‐231, BT‐549, MCF‐7, MDA‐MB‐468, BT‐474, T47D, SK‐BR‐3) relative to the non‐tumorigenic MCF‐10A cell line (Figure 1B,C). To corroborate these findings in clinical samples, we analyzed freshly resected tumors and matched adjacent normal tissues from four breast cancer patients treated at Chongqing University Cancer Hospital. Immunoblotting revealed consistently reduced FXYD1 protein levels in tumor tissues compared to normal controls (Figure 1D). In addition, immunohistochemical (IHC) analysis of FXYD1 in a cohort of 60 surgical breast cancer specimens from Chongqing University Cancer Hospital further confirmed significantly lower protein expression in tumor tissues (Figure 1E,F). Moreover, Kaplan‐Meier survival analysis demonstrated that low FXYD1 expression was significantly associated with reduced OS, relapse‐free survival (RFS), and distant metastasis‐free survival (DMFS) in KM‐Plotter dataset (Figure 1G–I). In our clinical cohort, Cox proportional hazards modeling further supported the prognostic value of FXYD1, as it was significantly associated with RFS in univariate analysis (Table S1) and remained an independent protective factor after multivariable adjustment (Table S2). Collectively, these multi‐dimensional results identify FXYD1 as a consistently downregulated gene in breast cancer and establish its reduced expression as a robust predictor of poor clinical outcomes.

### 2.2 Promoter Hypermethylation Induces FXYD1 Silencing in Breast Cancer

Previous studies have suggested a strong association between FXYD1 expression and DNA methylation [23]. To systematically investigate this relationship, we analyzed the methylation landscape of the FXYD1 promoter region across multiple cancer types using the SMART online tool. The analysis revealed significantly elevated promoter methylation of FXYD1 in breast cancer, as well as in several other malignancies (Figure S4A). Consistent with this observation, analysis of the TCGA cohort further showed an inverse correlation between FXYD1 promoter methylation (β values) and FXYD1 mRNA expression (Figure S4B). We next treated two breast cancer cell lines with low endogenous FXYD1 levels (MDA‐MB‐231 and BT‐549), along with the non‐tumorigenic MCF‐10A cell line, using the DNA methyltransferase inhibitor 5‐aza‐2’‐deoxycytidine (Aza) for 24 h. qRT‐PCR analysis revealed that Aza treatment led to a significant upregulation of FXYD1 expression in the methylated MDA‐MB‐231 and BT‐549 cells, whereas the non‐methylated MCF‐10A cells showed no appreciable change (Figure S4C). To further confirm the impact of promoter methylation in regulating FXYD1 expression, bisulfite sequencing PCR (BSP) was performed to evaluate the methylation status of the FXYD1 promoter in breast cancer cell lines and primary clinical tissues. Notably, a marked reduction in promoter methylation was observed in Aza‐treated cells, which correlated with the restoration of FXYD1 expression (Figure S4D). Consistently, in clinical samples (n = 3 pairs; Table S3), breast cancer tissues with low FXYD1 expression exhibited significant promoter hypermethylation compared to matched adjacent normal tissues (Figure S4E). Together, these results demonstrate that FXYD1 expression is epigenetically silenced in breast cancer through promoter CpG island hypermethylation, highlighting a potential mechanism underlying its transcriptional downregulation.

### 2.3 FXYD1 Suppresses Breast Cancer Cell Proliferation and Metastasis

**FIGURE 2:**
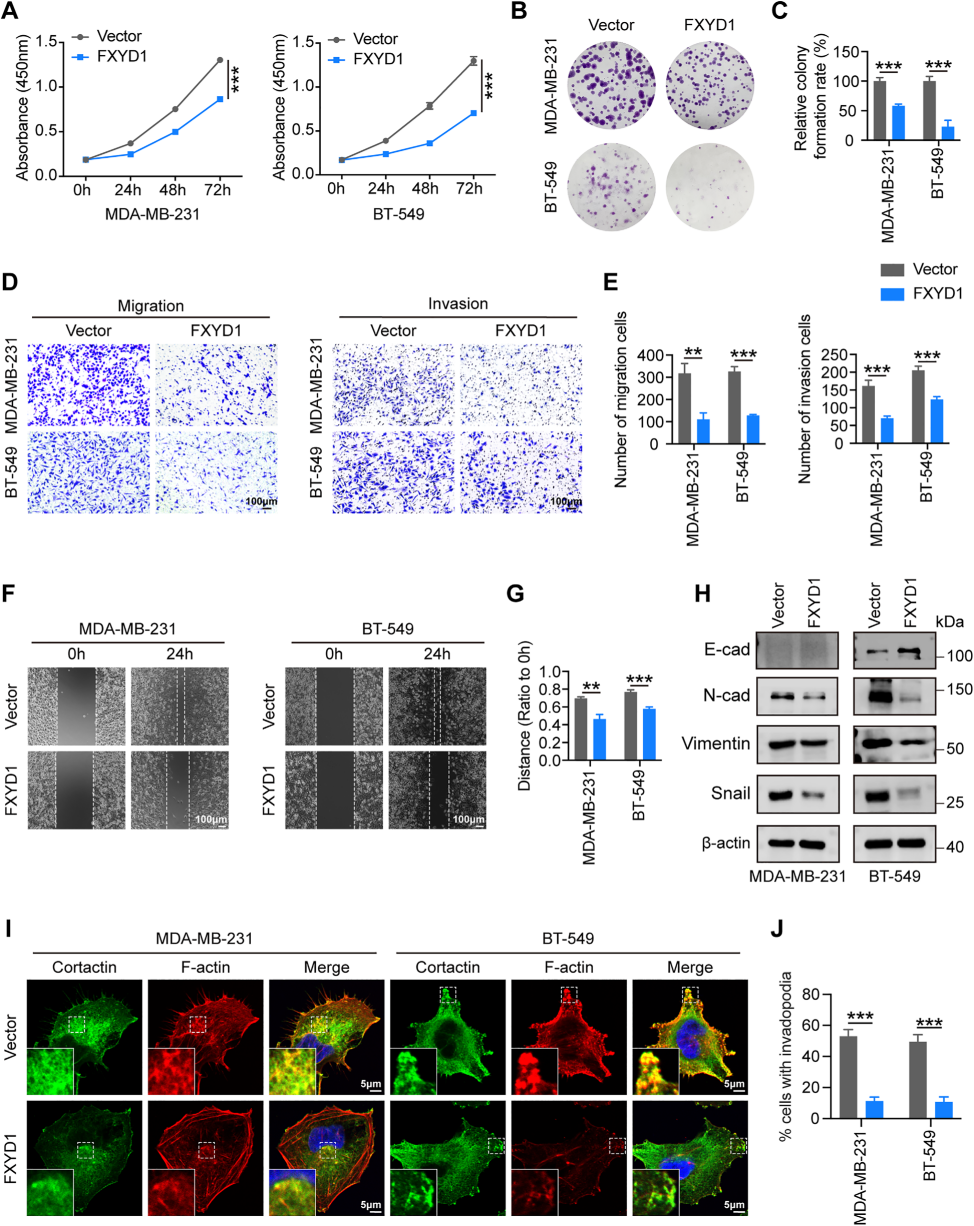
FXYD1 suppresses breast cancer cell proliferation and metastasis in vitro. (A–C) Cell proliferation was assessed by CCK‐8 assay (A) and colony formation assay (B, C). (D–G) The transwell (D, E) and wound‐healing (F, G) assays detecting the migration and invasion. (H) The expression levels of EMT‐related markers, including E‐cadherin, N‐cadherin, Vimentin, and the EMT‐associated transcription factor Snail detected by immunoblotting. E‐cadherin is intrinsically low in MDA‐MB‐231 cells; therefore, an E‐cadherin band is not detectable. (I, J) Representative images (I) and percentage of cells with invadopodia (J). Invadopodia is identified as co‐localization of cortactin (green) and F‐actin (red) in IF staining. Data are presented as the mean ± SD (n = 3 independent experiments). **p < 0.01, ***p < 0.001 vs. indicated controls in one‐sided unpaired t‐test.

To investigate the role of FXYD1 in breast cancer progression, we ectopically expressed FXYD1 in MDA‐MB‐231 and BT‐549 cells, which exhibit low endogenous levels of FXYD1, using lentiviral transduction. Conversely, FXYD1 expression was silenced in T47D cells, which have high endogenous FXYD1 expression, via siRNA‐mediated knockdown (Figure S5A,B). CCK‐8 and colony formation assays demonstrated that FXYD1 overexpression significantly inhibited the proliferation of MDA‐MB‐231 and BT‐549 cells, whereas FXYD1 knockdown markedly promoted proliferation in T47D cells (Figure 2A–C; Figure S5C). Notably, overexpression of FXYD1 also significantly impaired the migratory and invasive abilities of MDA‐MB‐231 and BT‐549 cells, as evidenced by reduced cell migration/invasion in transwell and wound‐healing assays (Figure 2D–G). These phenotypic changes were further supported by molecular analyses, which showed increased expression of the epithelial marker E‐cadherin and decreased levels of mesenchymal markers N‐cadherin, Vimentin, and the EMT‐associated transcription factor Snail upon FXYD1 overexpression, as assessed by immunoblotting (Figure 2H). Immunofluorescence (IF) staining also revealed a reduced proportion of cells exhibiting invadopodia formation (Figure 2I,J). In contrast, FXYD1 knockdown in T47D cells enhanced migration, invasion, and EMT markers expression (Figure S5D–H).

**FIGURE 3:**
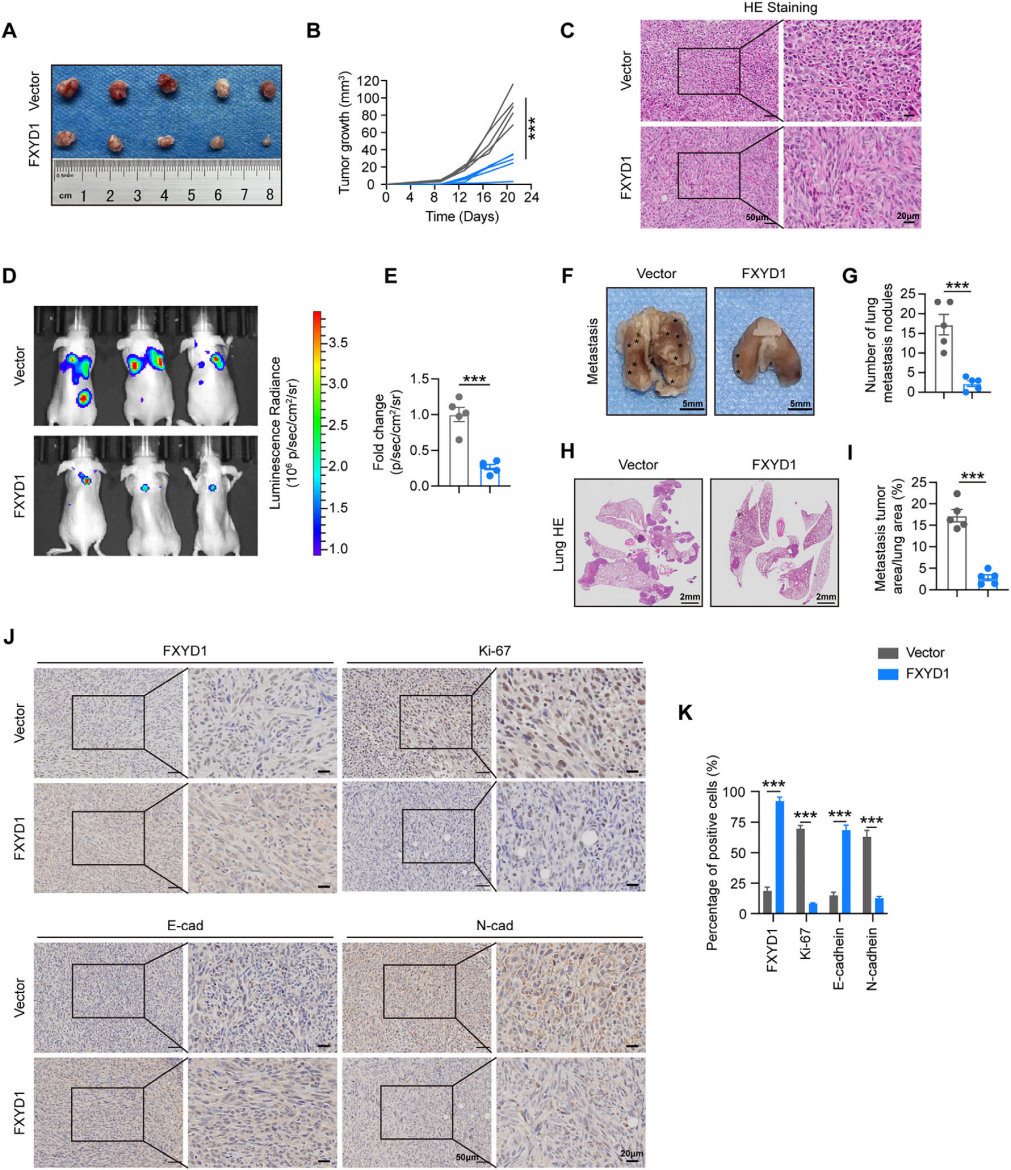
FXYD1 suppresses breast cancer cell proliferation and metastasis in vivo. (A, B) Vector or FXYD1 overexpression MDA‐MB‐231 cells were subcutaneously inoculated into nude mice. Tumor image (A) and tumor growth curves (B) are shown. Data are presented as the mean ± SD (n = 5 mice). (C) Representative HE staining of tumor sections in mice. (D, E) Representative bioluminescent images of lung metastasis model at day 28 after tail vein injection (D) and quantification of lung luminescence radiance normalized to the vector group (E). (F–I) The lung metastasis foci (F) and the HE staining of lung sections (H) in the lung metastasis model. Metastatic foci and areas are indicated by asterisks (*). Quantification of the number of metastatic nodules (G) and percentage of metastatic area (I) in lung sections. (J, K) Representative images (J) and quantification (K) of IHC staining for FXYD1, Ki‐67, E‐cadherin, and N‐cadherin in xenograft tumors. Data are presented as the mean ± SD. ***p < 0.001 vs. indicated controls in one‐sided unpaired t‐test.

To further evaluate the tumor‐suppressive role of FXYD1 in vivo, we established subcutaneous xenograft and lung metastasis models by injecting MDA‐MB‐231 cells stably expressing FXYD1 or vector control into the flank of female nude mice. As expected, FXYD1 overexpression significantly reduced tumor growth rate and final tumor volume in the xenograft model (Figure 3A–C), and markedly decreased the number of metastatic nodules in the lung metastasis model (Figure 3D–I). Moreover, IHC analysis of xenograft tumors revealed decreased expression of the proliferation marker Ki‐67 and the mesenchymal marker N‐cadherin, along with increased expression of E‐cadherin, in the FXYD1‐overexpressing group compared to controls (Figure 3J,K). Thus, these findings demonstrate that FXYD1 acts as a potent suppressor of breast cancer cell proliferation and metastasis both in vitro and in vivo.

### 2.4 FXYD1 Inhibits Tumor Progression by Attenuating Wnt/β‐Catenin Signaling

**FIGURE 4:**
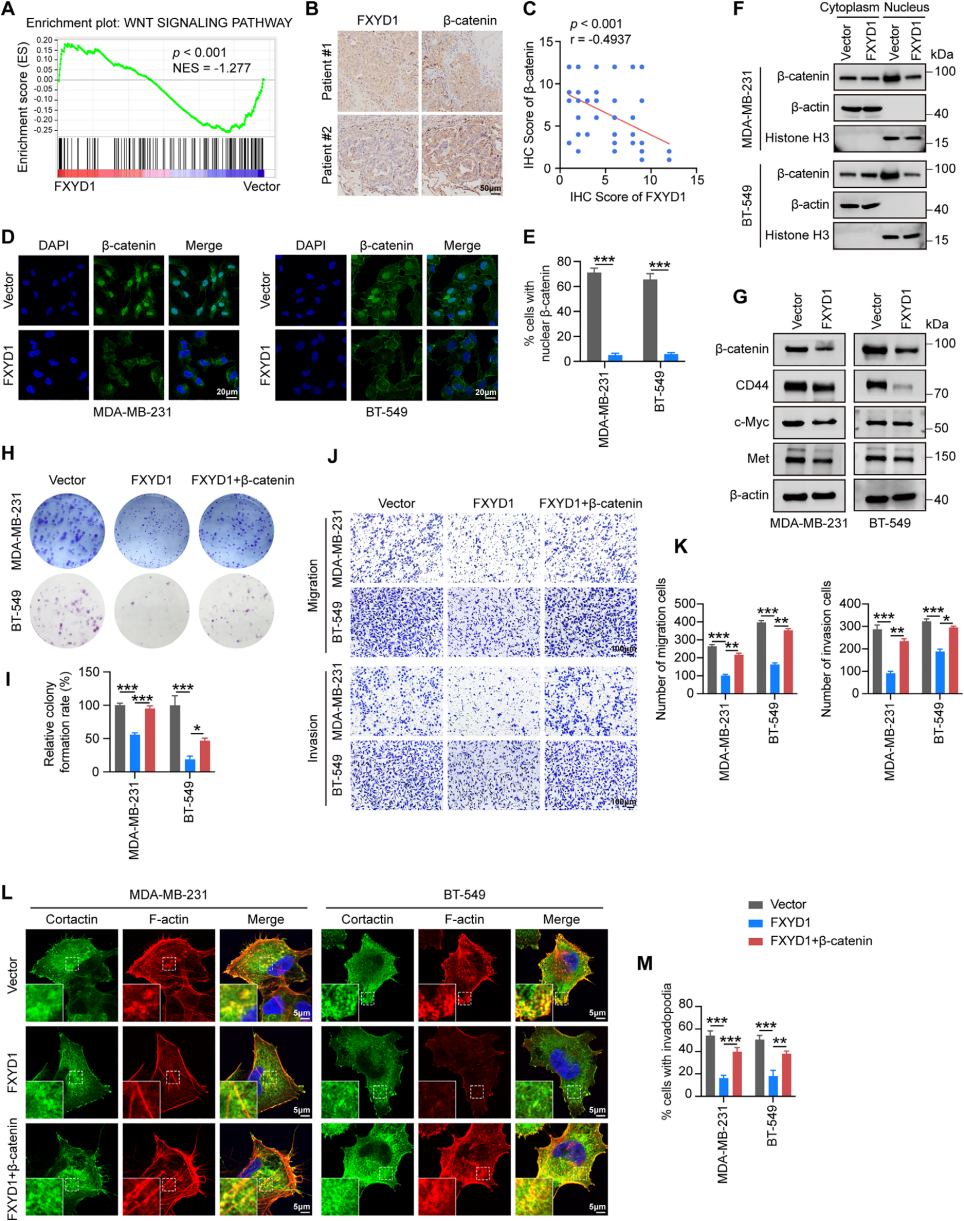
FXYD1 inhibits tumor progression by attenuating Wnt/β‐catenin signaling. (A) GSEA plots showing downregulation of Wnt/β‐catenin pathway genes in FXYD1 overexpressing MDA‐MB‐231 cells. Statistical analysis was performed using GSEA. (B) Representative IHC images of human breast cancer tissues with high or low FXYD1 expression and corresponding low or high β‐catenin expression (n = 53 samples). (C) Two‐tailed Pearson correlation test analyzing the relationship between the IHC staining scores of FXYD1 and β‐catenin in breast cancer tissues (n = 53 samples). (D, E) IF staining (D) and quantification (E) for nuclear β‐catenin in MDA‐MB‐231 and BT‐549 cells transduced with vector or FXYD1. One‐sided unpaired t‐test. (F) Immunoblot analysis of cytoplasmic and nuclear fractions showing reduced nuclear β‐catenin upon FXYD1 overexpression. (G) Immunoblot analysis of β‐catenin, CD44, c‐Myc, and Met in MDA‐MB‐231 and BT‐549 cells transduced with vector or FXYD1. (H–M) Functional assays evaluating cell proliferation, migration, invasion, and invadopodia formation in MDA‐MB‐231 and BT‐549 cells with vector, FXYD1 overexpression, or FXYD1 plus β‐catenin co‐expression, including colony formation (H, I), transwell (J, K), and IF (L, M) assays. One‐way ANOVA. Data are presented as the mean ± SD (n = 3 independent experiments). *p < 0.05, **p < 0.01, ***p < 0.001.

To elucidate the molecular mechanism by which FXYD1 inhibits breast cancer progression, we performed RNA sequencing on MDA‐MB‐231 cells stably overexpressing FXYD1 or vector control. Gene Set Enrichment Analysis (GSEA) revealed a significant downregulation of the Wnt/β‐catenin signaling pathway in FXYD1‐overexpressing cells (Figure 4A). Supporting this, IHC analysis of 53 human breast cancer samples showed a strong inverse correlation between FXYD1 and β‐catenin protein levels (Figure 4B,C). Consistently, xenograft tumors derived from FXYD1‐overexpressing cells exhibited reduced β‐catenin staining compared with controls (Figure S6A,B). Given the well‐established role of Wnt/β‐catenin signaling in promoting tumor proliferation and metastasis, we hypothesized that FXYD1 may exert its tumor‐suppressive effects by inhibiting this pathway. IF staining and nuclear/cytoplasmic fractionation assays demonstrated a substantial reduction in nuclear localization of β‐catenin in FXYD1‐overexpressing cells compared to controls, where β‐catenin exerts its transcriptional activity (Figure 4D–F; Figure S6C). Consistently, immunoblotting and qRT‐PCR showed that ectopic expression of FXYD1 markedly reduced total β‐catenin levels as well as the expression of downstream targets, including c‐Myc, CD44, and Met. Conversely, FXYD1 knockdown increased β‐catenin levels and upregulated these target genes (Figure 4G; Figure S6D–F), indicating a negative regulatory relationship between FXYD1 and Wnt/β‐catenin signaling in breast cancer cells.

To determine whether β‐catenin mediates the tumor‐suppressive function of FXYD1, we conducted rescue experiments by reintroducing β‐catenin into FXYD1‐overexpressing MDA‐MB‐231 and BT‐549 cells. Strikingly, restoration of β‐catenin expression largely reversed the inhibitory effects of FXYD1 on cell proliferation, migration, and invasion, as evidenced by increased colony formation, enhanced migratory and invasive capacities, and a higher proportion of invadopodia‐positive cells (Figure 4H–M; Figure S6G). Consistently, qRT‐PCR analyses showed that Wnt/β‐catenin downstream target genes were robustly upregulated upon β‐catenin re‐expression in the FXYD1‐overexpressing context (Figure S6H). Collectively, these results confirm that inhibition of Wnt/β‐catenin signaling is a key mechanism by which FXYD1 suppresses breast cancer progression.

### 2.5 FXYD1 Mediates DDX5 Ubiquitination at Lysine 470 in Breast Cancer Cells

**FIGURE 5:**
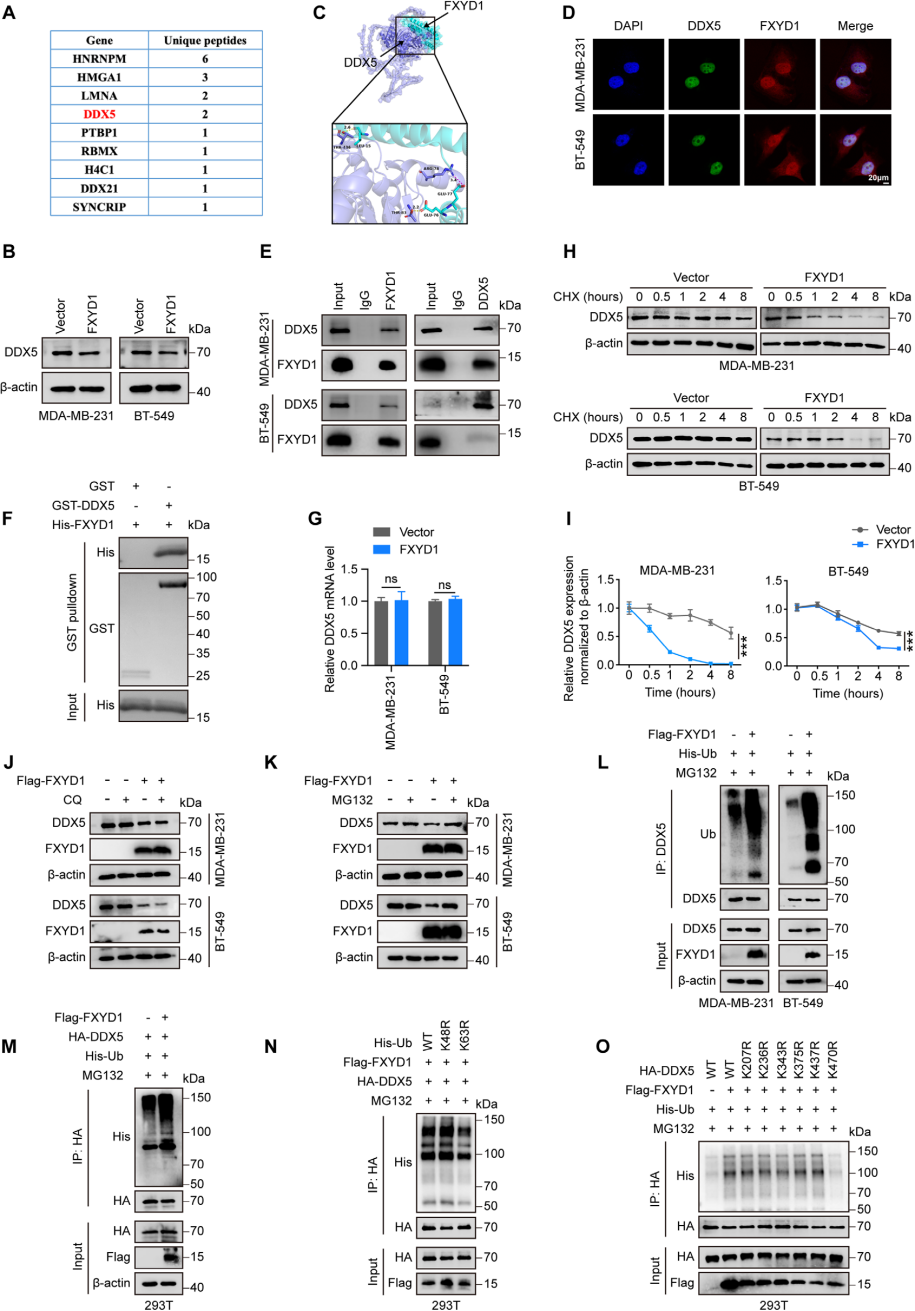
FXYD1 mediates DDX5 ubiquitination at lysine 470 in breast cancer cells. (A) The top 10 FXYD1‐interacting proteins identified by MS. (B) Immunoblot analysis of DDX5 expression in MDA‐MB‐231 and BT‐549 cells transduced with Vector or FXYD1. (C) 3D structural modeling predicting the direct association between FXYD1 and DDX5. (D) IF analysis showing nuclear colocalization of FXYD1 (red) and DDX5 (green) in MDA‐MB‐231 and BT‐549 cells. (E) Co‐IP assays detecting the interaction between endogenous FXYD1 and DDX5 in MDA‐MB‐231 and BT‐549 cells. (F) GST pulldown assays confirming the direct binding between recombinant FXYD1 and DDX5. Recombinant GST‐DDX5 protein was incubated with recombinant His‐FXYD1 protein, followed by GST pulldown and immunoblotting analysis with GST and His antibodies. (G) Relative mRNA level of DDX5 in FXYD1 overexpression cells. (H) Immunoblotting analysis of DDX5 protein stability in FXYD1 overexpression cells treated with or without CHX (50 µg/mL) for the indicated time periods. (I) Quantification of DDX5 protein levels based on immunoblot band intensities. (J) Immunoblot analysis of DDX5 in FXYD1 overexpression cells treated with or without CQ (40 µm, 8 h). (K) Immunoblot analysis of DDX5 in FXYD1‐overexpressing cells treated with or without MG132 (20 µm, 8 h). (L) Vector‐ or FXYD1‐overexpressing MDA‐MB‐231 and BT‐549 cells transduced with His‐ubiquitin (His‐Ub) were treated with MG132 (20 µm, 8 h). DDX5 was immunoprecipitated, and ubiquitination was assessed by immunoblotting with an anti‐ubiquitin antibody. (M) HEK293T cells were co‐transfected with Flag‐FXYD1, HA‐DDX5, and His‐Ub for 48 h, followed by MG132 treatment (20 µm, 8 h). The ubiquitination level of DDX5 was detected by immunoprecipitated. (N) HEK293T cells were co‐transfected with Flag‐FXYD1, HA‐DDX5, and either His‐Ub‐WT, His‐Ub‐K48R, or His‐Ub‐K63R for 48 h, followed by MG132 (20 µm, 8 h) treatment. DDX5 ubiquitination was analyzed by immunoprecipitation with HA antibody and immunoblotting. (O) HEK293T cells were co‐transfected with HA‐DDX5 (WT or the indicated KR mutants), His‐Ub, with or without Flag‐FXYD1. After MG132 (20 µm, 8 h) treatment, HA‐DDX5 was immunoprecipitated with HA antibody, and ubiquitination was assessed by immunoblotting with the indicated antibodies. Data are presented as the mean ± SD (n = 3 independent experiments). ns, not significant, ***p < 0.001 vs. indicated controls in one‐sided unpaired t‐test.

We next sought to elucidate how FXYD1 regulates the Wnt/β‐catenin signaling pathway. To identify downstream effectors of FXYD1 in breast cancer cells, we performed immunoprecipitation using an anti‐Flag antibody in MDA‐MB‐231 cells stably expressing Flag‐tagged FXYD1, followed by Mass spectrometry (MS) analysis. This analysis identified 27 candidate FXYD1‐interacting proteins. Among them, four proteins‐HNRNPM, HMGA1, LMNA, and DDX5‐were detected with two or more unique peptides (Figure 5A). Previous studies have reported that HNRNPM, HMGA1, and DDX5 are frequently upregulated in breast cancer and are associated with poor clinical outcomes, whereas high LMNA expression is linked to favorable prognosis and decreased malignancy [29, 30, 31, 32]. Among these, only DDX5 was significantly downregulated by FXYD1 (Figure 5B; Figure S7A,B). This is consistent with prior evidence that DDX5 binds to β‐catenin, enhancing its transcriptional activity and promoting expression of downstream oncogenic targets [33, 34]. Based on these findings, we hypothesized that DDX5 may be a critical mediator of FXYD1‐dependent regulation of Wnt/β‐catenin signaling.

To test this hypothesis, we first conducted molecular docking analysis to assess the interaction potential between FXYD1 and DDX5 at the protein–protein interface. Encouragingly, molecular docking simulations predicted a strong interaction between FXYD1 and DDX5 (Figure 5C). Consistently, IF staining showed that FXYD1 and DDX5 co‐localized within the nucleus of MDA‐MB‐231 and BT‐549 cells (Figure 5D). This interaction was further validated by co‐immunoprecipitation (Co‐IP) assays using both endogenous and ectopically expressed proteins (Figure 5E; Figure S7C). Moreover, GST pulldown assays confirmed that FXYD1 directly binds to DDX5 (Figure 5F). Thus, these results indicate that FXYD1 directly binds to and downregulates DDX5 in breast cancer cells. Notably, qRT‐PCR analysis showed that neither overexpression nor knockdown of FXYD1 altered DDX5 mRNA levels in breast cancer cells (Figure 5G; Figure S7D), thereby excluding transcriptional regulation. Given above results, we hypothesized that FXYD1 modulates DDX5 expression through a post‐transcriptional mechanism. Consistent with this, protein stability assays revealed that FXYD1 overexpression significantly reduced the half‐life of DDX5 in breast cancer cells (Figure 5H,I). Moreover, treatment with the proteasome inhibitor MG132, but not the lysosomal inhibitor chloroquine (CQ), effectively rescued FXYD1‐induced DDX5 downregulation (Figure 5J,K), indicating that FXYD1 promotes proteasome‐dependent degradation of DDX5. Supporting this notion, FXYD1 overexpression markedly enhanced the ubiquitination of both exogenous DDX5 in HEK293T cells and endogenous DDX5 in MDA‐MB‐231 and BT‐549 cells (Figure 5L,M).

Ubiquitin chains linked through different lysine residues exert distinct functional consequences: K48‐linked polyubiquitination primarily targets proteins for proteasomal degradation, whereas K63‐linked chains are generally involved in non‐proteolytic processes such as signaling and protein trafficking [35, 36, 37]. To identify the specific linkage involved in FXYD1‐mediated ubiquitination of DDX5, HEK293T cells were transfected with either His‐K48R or His‐K63R ubiquitin mutants. Remarkably, FXYD1‐induced DDX5 ubiquitination was abolished in cells expressing the K63R mutant but remained unaffected in those expressing the K48R mutant (Figure 5N), suggesting that FXYD1 facilitates K63‐linked ubiquitination of DDX5. To further identify the target lysine residues on DDX5, we performed LC‐MS/MS analysis on immunoprecipitated HA‐DDX5, revealing six candidate ubiquitination sites: K207, K236, K343, K375, K437, and K470. Site‐directed mutagenesis was then used to individually substitute these lysines with arginine, followed by in vivo ubiquitination assays. Interestingly, FXYD1 overexpression significantly increased the ubiquitination of wild‐type HA‐DDX5 and all mutant variants except K470R (Figure 5O), indicating that K470 is essential for FXYD1‐mediated ubiquitination of DDX5. Collectively, these findings demonstrate that, FXYD1 directly binds to DDX5 and promotes K63‐linked ubiquitination of DDX5 at lysine 470 in breast cancer cells.

### 2.6 FXYD1 Targets DDX5 to Inhibit Activation of Wnt/β‐Catenin Signaling

**FIGURE 6:**
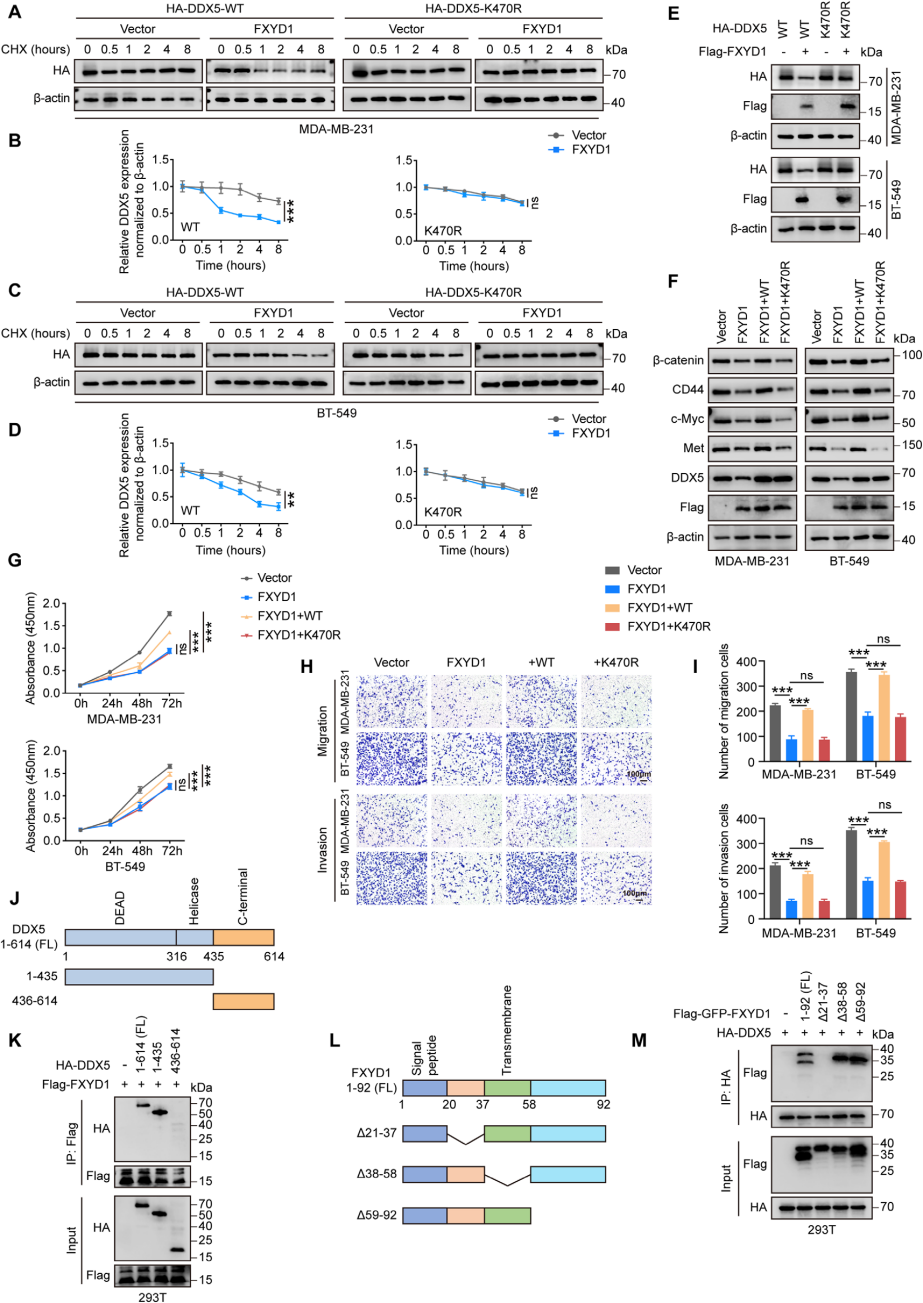
FXYD1 targets DDX5 to inhibit activation of Wnt/β‐catenin signaling. (A–D) FXYD1 overexpression cells were transfected with HA‐DDX5 WT or K470R plasmid, and treated with or without CHX (50 µg/mL) for the indicated time. Immunoblotting analysis of HA‐DDX5 was performed (A, C). Quantification of HA‐DDX5 protein levels based on immunoblot band intensities (B, D). One‐sided unpaired t‐test. (E) Flag‐FXYD1 was co‐expressed with HA‐DDX5 WT or K470R in MDA‐MB‐231 and BT‐549 cells, followed by immunoblotting. (F–I) Functional assays assessing the role of DDX5 in mediating the effects of FXYD1. MDA‐MB‐231 and BT‐549 cells transduced with Vector or FXYD1, as well as FXYD1‐overexpressing cells further transduced with DDX5‐WT or K470R, were analyzed for Wnt/β‐catenin signaling activity, cell proliferation, migration, and invasion using immunoblotting (F), CCK‐8 assays (G), and transwell assays (H, I). One‐way ANOVA. (J) Schematic representation of full‐length (FL) DDX5 and its truncation mutants. (K) HEK293T cells were co‐transfected with HA‐DDX5 FL or the indicated truncation mutants together with Flag‐FXYD1. Flag‐FXYD1 was immunoprecipitated with Flag antibody, and associated HA‐DDX5 was detected by immunoblotting with HA antibody. (L) Schematic representation of FL FXYD1 and its truncation mutants. (M) HEK293T cells were co‐transfected with HA‐DDX5 and FL Flag‐GFP‐FXYD1 or the indicated truncation mutants. HA‐DDX5 was immunoprecipitated with HA antibody, followed by immunoblotting analysis of Flag‐GFP‐FXYD1 with Flag antibody. Data are presented as the mean ± SD (n = 3 independent experiments). ns, not significant, **p < 0.01, ***p < 0.001.

To examine whether lysine 470 contributes to DDX5 turnover, we compared the stability of WT‐DDX5 and the K470R mutant. Notably, overexpression of FXYD1 substantially shortened the half‐life of WT‐DDX5, whereas it had little to no effect on the stability of DDX5‐K470R (Figure 6A–D). Consistently, FXYD1 led to an apparent decrease in the level of WT‐DDX5 but had no obvious effect on the K470R mutant (Figure 6E), indicating that K470 is a critical determinant for FXYD1‐driven DDX5 degradation.

We next asked whether attenuation of Wnt/β‐catenin signaling by FXYD1 depends on its regulation of DDX5. To this end, we performed rescue experiments by re‐expressing DDX5 in FXYD1‐overexpressing MDA‐MB‐231 and BT‐549 cells. FXYD1 overexpression reduced β‐catenin abundance and diminished its nuclear accumulation; both effects were robustly restored by re‐expression of WT‐DDX5, but not by DDX5‐K470R (Figure 6F; Figure S7E–G). In line with these molecular changes, WT‐DDX5—but not DDX5‐K470R—rescued the FXYD1‐induced suppression of cell proliferation, migration, and invasion (Figure 6G–I; Figure S7H–J). Together with the resistance of DDX5‐K470R to FXYD1‐induced ubiquitination and turnover, these data establish K470 as a functionally essential ubiquitination site required for DDX5 regulation within the FXYD1–DDX5 axis and for the associated Wnt/β‐catenin–dependent malignant phenotypes.

To delineate the interaction domain of DDX5 required for binding FXYD1, we constructed two HA‐tagged truncation mutants: one lacking the N‐terminal DEAD and Helicase domains (1‐435aa), and the other lacking the C‐terminal region (436‐614aa). These constructs were co‐expressed with Flag‐FXYD1 in HEK293T cells (Figure 6J). Co‐IP assays revealed that the N‐terminal region of DDX5 is required for its interaction with FXYD1 (Figure 6K). We then constructed FXYD1 deletion mutants lacking the N‐terminal (21‐37aa), transmembrane (38‐58aa), or C‐terminal (59‐92aa) domains and co‐expressed them with HA‐DDX5 in HEK293T cells (Figure 6L). Co‐IP assays revealed that disruption of the N‐terminal region of FXYD1 abolished its binding to DDX5, indicating that this region is required for the interaction (Figure 6M). Collectively, these findings support a model in which FXYD1 engages DDX5 through their N‐terminal regions and promotes K470‐dependent ubiquitination and degradation of DDX5, thereby dampening Wnt/β‐catenin signaling and inhibiting breast cancer cell proliferation and metastasis.

### 2.7 FXYD1 Scaffolds MAEA to Facilitate DDX5 Ubiquitination

**FIGURE 7:**
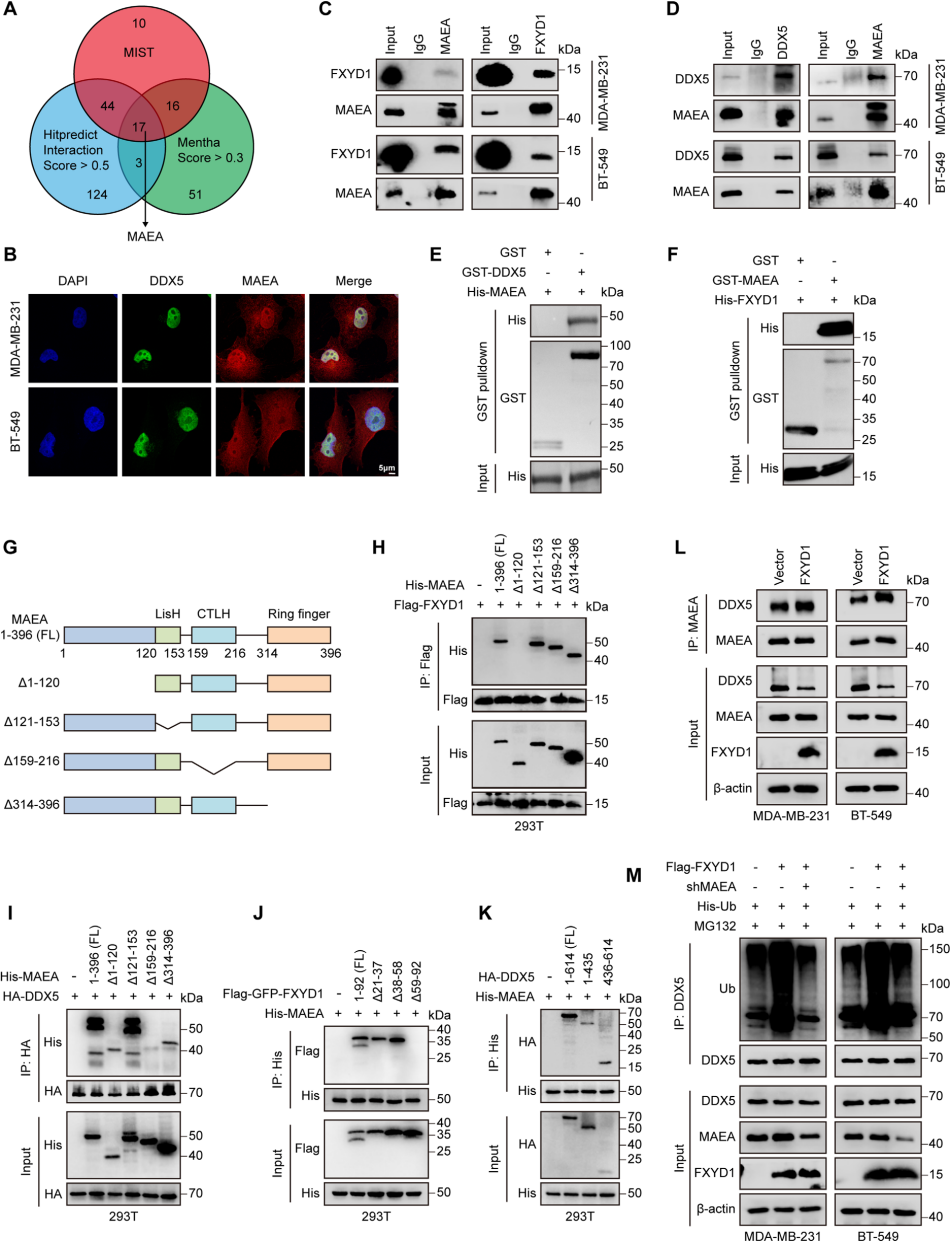
FXYD1 scaffolds MAEA to facilitate DDX5 ubiquitination. (A) Venn diagram showing the identification of DDX5 binding partners predicted by MIST, Hitpredict, and Mentha protein interaction databases. (B) IF analysis showing nuclear colocalization of MAEA (red) and DDX5 (green) in MDA‐MB‐231 and BT‐549 cells. (C, D) Co‐IP assays detecting the interaction between endogenous MAEA and FXYD1 (C) or DDX5 (D) in MDA‐MB‐231 and BT‐549 cells. (E–F) GST pulldown assays confirming direct binding of recombinant DDX5 with MAEA (E) and recombinant FXYD1 with MAEA (F). Recombinant GST fusion proteins were incubated with the indicated His‐tagged proteins, followed by GST pulldown and immunoblotting with anti‐GST and anti‐His antibodies. (G) Schematic representation of FL MAEA and the indicated truncation mutants. (H) HEK293T cells were co‐transfected with His‐MAEA (FL or the indicated truncation mutants) and Flag‐FXYD1. Flag‐FXYD1 was immunoprecipitated with anti‐Flag antibody, and associated His‐MAEA was detected by immunoblotting with anti‐His antibody. (I) HEK293T cells were co‐transfected with His‐MAEA (FL or the indicated truncation mutants) and HA‐DDX5. HA‐DDX5 was immunoprecipitated with anti‐HA antibody, and associated His‐MAEA was detected by immunoblotting with anti‐His antibody. (J) HEK293T cells were co‐transfected with Flag‐GFP‐FXYD1 (FL or the indicated truncation mutants) and His‐MAEA. His‐MAEA was immunoprecipitated with anti‐His antibody, and bound Flag‐GFP‐FXYD1 was detected by immunoblotting with anti‐Flag antibody. (K) HEK293T cells were co‐transfected with HA‐DDX5 (FL or the indicated truncation mutants) and His‐MAEA. His‐MAEA was immunoprecipitated with anti‐His antibody, and bound HA‐DDX5 was detected by immunoblotting with anti‐HA antibody. (L) Co‐IP assays examining the interaction between MAEA and DDX5 in MDA‐MB‐231 and BT‐549 cells with or without FXYD1 overexpression. (M) His‐Ub was co‐expressed with Flag‐FXYD1 in MAEA knockdown cells. After MG132 (20 µm, 8 h) treatment, DDX5 was immunoprecipitated with anti‐DDX5 antibody, and ubiquitination was assessed by immunoblotting with the indicated antibodies.

As FXYD1 lacks intrinsic enzymatic activity required for ubiquitin‐mediated protein degradation, we hypothesized that it may exert its regulatory function by recruiting specific E3 ubiquitin ligases or deubiquitinases to promote DDX5 ubiquitination and subsequent degradation. To investigate this possibility, we searched the MIST, HitPredict, and Mentha protein interaction databases for potential DDX5‐binding partners (Figure 7A), identifying 17 high‐confidence candidate interactors. Notably, MAEA was the only E3 ubiquitin ligase among these candidates, suggesting it may serve as a functional link between FXYD1 and DDX5.

Supporting this hypothesis, IF analysis revealed that DDX5 and MAEA co‐localize within the nucleus of MDA‐MB‐231 and BT‐549 cells, closely resembling the nuclear co‐localization pattern previously observed between FXYD1 and DDX5 (Figure 5D and Figure 7B). Moreover, Co‐IP assays confirmed that MAEA interacts with both DDX5 and FXYD1 under endogenous and ectopically expressed conditions (Figure 7C,D; Figure S8A,B). This physical interaction was further validated by GST pulldown assays, which demonstrated direct binding of MAEA to both FXYD1 and DDX5 (Figure 7E,F). Together with our prior evidence demonstrating a direct interaction between FXYD1 and DDX5, these findings strongly support the formation of a ternary complex composed of FXYD1, MAEA, and DDX5 in breast cancer cells.

We next mapped the domains of MAEA required for its interaction with FXYD1 and DDX5, respectively. To this end, we generated MAEA deletion mutants lacking the N‐terminal (1‐120aa), LisH (121‐153aa), CTLH (159‐216aa), or RING finger (314‐396aa) domains, and co‐expressed them with Flag‐FXYD1 or HA‐DDX5 in HEK293T cells (Figure 7G). Co‐IP assays revealed that MAEA interacts with FXYD1 via its N‐terminal domain (Figure 7H), whereas it binds to DDX5 through its CTLH domain (Figure 7I). Furthermore, by co‐transducing full‐length MAEA with various deletion mutants of FXYD1 or DDX5 in HEK293T cells, we found that MAEA associates with the C‐terminal domain of FXYD1 (Figure 7J) and the C‐terminal region of DDX5 exhibited higher affinity to MAEA (Figure 7K). Taken together with our previous results (Figure 6J–M), these findings demonstrate that the N‐terminal domain of FXYD1 binds to the N‐terminal domain of DDX5, while MAEA interacts with the C‐terminal domains of both FXYD1 and DDX5 through its N‐terminal and CTLH domains, respectively, thereby stabilizing the formation of the FXYD1–MAEA–DDX5 ternary complex.

**FIGURE 8:**
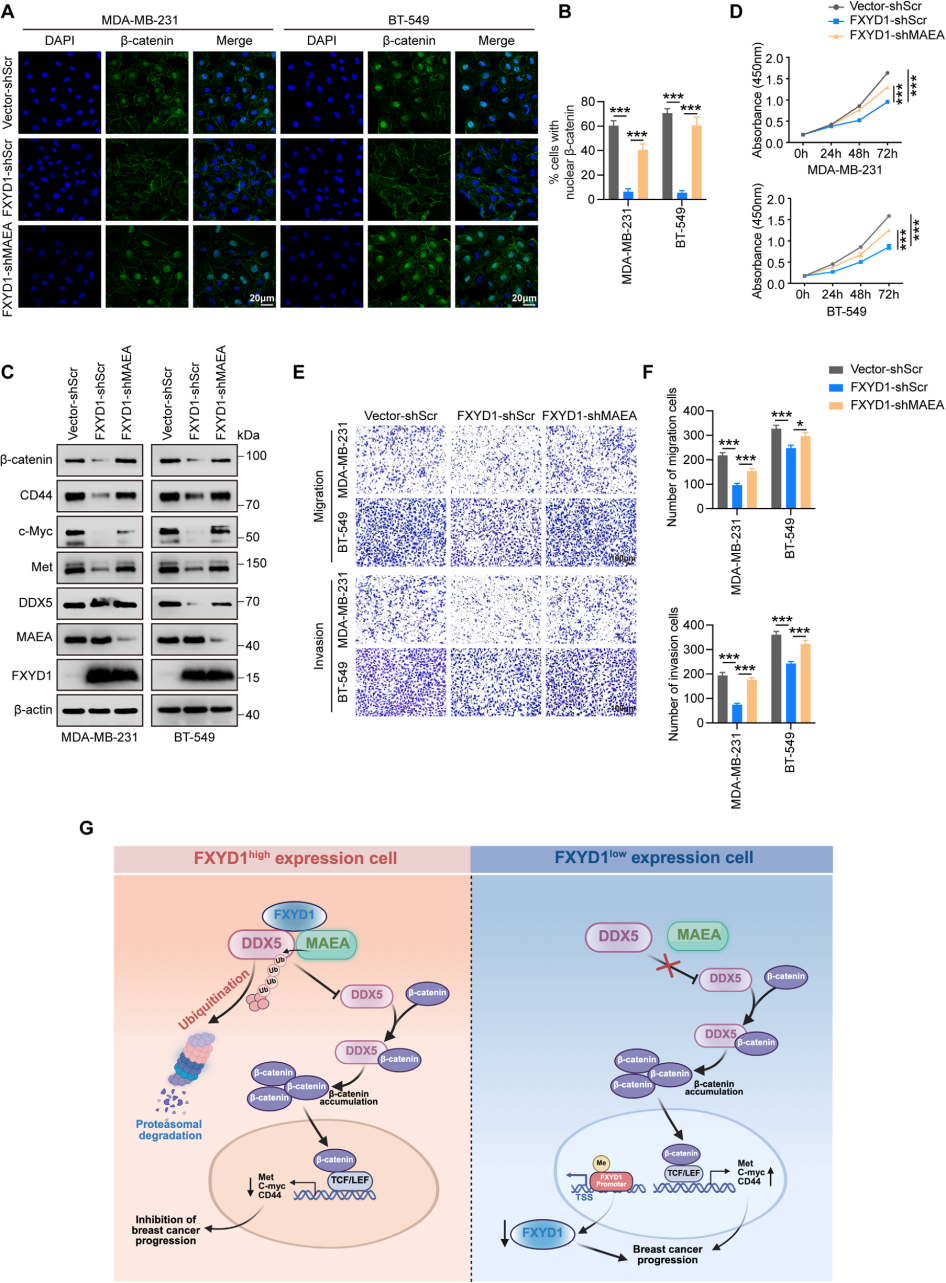
MAEA is involved in FXYD1‐mediated suppression of breast cancer proliferation and metastasis. (A–F) MDA‐MB‐231 and BT‐549 cells with vector or FXYD1 overexpression were transduced with MAEA shRNA or scrambled shRNA control. Wnt/β‐catenin signaling activity, cell proliferation, migration, and invasion were assessed by IF (A, B), immunoblotting (C), CCK‐8 assays (D), and transwell assays (E, F). (G) Schematic model illustrating the FXYD1–MAEA–DDX5–β‐catenin axis in regulating breast cancer cell proliferation and metastasis. Data are presented as the mean ± SD (n = 3 independent experiments). *p < 0.05, ***p < 0.001 vs. indicated controls in one‐way ANOVA.

To further elucidate the regulatory relationship among these proteins, we examined how FXYD1 influences the interaction between MAEA and DDX5. Interestingly, Co‐IP assays revealed that overexpression of FXYD1 significantly facilitated or stabilized the interaction between MAEA and DDX5 compared to control cells (Figure 7L). Additionally, ubiquitination assays indicated that silencing MAEA via lentivirus‐mediated shRNA completely abolished FXYD1‐induced DDX5 ubiquitination and subsequent degradation in both MDA‐MB‐231 and BT‐549 cells (Figure 7M). We then assessed whether MAEA E3 ligase activity is required for this process. Reconstitution of MAEA‐depleted cells with WT‐MAEA, but not with the ligase‐dead mutant MAEA‐Y394A [38, 39], restored K63‐linked ubiquitination of DDX5 when a K63‐only ubiquitin construct was used (Figure S8C). These results establish that MAEA catalytic activity is indispensable for FXYD1‐driven K63‐linked ubiquitination of DDX5. Functionally, knockdown of MAEA reversed the suppressive effects of FXYD1 overexpression on Wnt/β‐catenin signaling activity (Figure 8A–C; Figure S8D), as well as on cell proliferation (Figure 8D), migration, and invasion (Figure 8E,F; Figure S8E–G) in breast cancer cells.

In summary, our data demonstrate that FXYD1 functions as a scaffold protein that enhances the binding of the E3 ubiquitin ligase MAEA to DDX5, thereby promoting DDX5 ubiquitination and proteasomal degradation, which in turn attenuates Wnt/β‐catenin signaling and suppresses the proliferative and metastatic potential of breast cancer cells (Figure 8G).

## 3 Discussion

In this study, we identified FXYD1 as a novel tumor suppressor in breast cancer and systematically elucidated its functional and mechanistic roles in inhibiting breast cancer progression. We demonstrate that FXYD1 is significantly downregulated in breast cancer, primarily due to promoter hypermethylation, and, in contrast to other FXYD family members, serves as a favorable prognostic marker. Mechanistically, FXYD1 functions as a scaffold that facilitates the recruitment of the E3 ubiquitin ligase MAEA to the RNA helicase DDX5, a key coactivator of β‐catenin. This interaction promotes K63‐linked polyubiquitination and subsequent proteasomal degradation of DDX5, thereby suppressing Wnt/β‐catenin signaling and inhibiting Wnt‐driven tumor growth and metastasis. These findings provide new insights into the biology of FXYD1 and underscore its unique role within the FXYD protein family, highlighting its potential as both a prognostic biomarker and a therapeutic target in breast cancer.

The FXYD family comprises seven members that encode a group of small membrane proteins characterized by a conserved FXYD motif, best known for their interaction with Na^+^/K^+^‐ATPase. Notably, our data show that FXYD1 is uniquely and consistently downregulated in breast cancer among all family members, and its reduced expression is strongly associated with poor patient outcomes. In contrast, other members such as FXYD3 and FXYD5 are frequently overexpressed in various malignancies, including breast and pancreatic cancers, and have been linked to increased cell motility and metastatic potential [18, 20, 21]. This divergent expression pattern suggests a context‐dependent functional specialization within the FXYD family. One possible explanation for these differences lies in subcellular localization. Remarkably, although FXYD proteins are classically described as small single‐pass membrane regulators of Na^+^/K^+^‐ATPase activity, our IF analyses revealed that FXYD1 is predominantly enriched in the nucleus and exhibits strong nuclear co‐localization with DDX5 (Figure 5D). This subcellular distribution provides an important spatial framework for understanding how FXYD1 exerts tumor‐suppressive functions beyond canonical ion homeostasis. DDX5 is a predominantly nuclear DEAD‐box helicase with well‐established roles in transcriptional regulation and oncogenic signaling, and prior studies have linked DDX5 to Wnt/β‐catenin–dependent transcriptional programs, including a reported positive feedback loop between β‐catenin/TCF4 signaling and DDX5 expression in breast cancer [33]. Therefore, the nuclear pool of FXYD1 may be functionally critical by positioning FXYD1 in proximity to nuclear DDX5 and enabling efficient coupling of ubiquitin‐dependent regulation of DDX5 to downstream transcriptional outputs of the Wnt/β‐catenin pathway. In this context, our mechanistic findings support a model in which nuclear FXYD1 acts as a scaffold that facilitates MAEA‐dependent ubiquitin signaling on DDX5, thereby restraining β‐catenin–driven nuclear transcriptional activity.

Notably, the presence of membrane‐associated proteins within the nucleus is increasingly appreciated as a biologically meaningful phenomenon rather than an experimental artifact. Canonical examples include receptor tyrosine kinases such as EGFR, whose nuclear localization has been implicated in direct transcriptional regulation and oncogenic phenotypes [40]. In addition, multiple routes have been described for how membrane‐tethered proteins can access the nucleus, including regulated proteolysis/release from membranes, retrograde trafficking, and importin‐dependent nuclear import of released cytosolic domains [41]. Although our current study does not yet delineate the trafficking mechanism that drives FXYD1 nuclear enrichment, these precedents raise the possibility that post‐translational modifications and binding partners could facilitate FXYD1 nuclear targeting and thereby drive its non‐canonical tumor‐suppressive function. Future work will be directed toward mapping the determinants of FXYD1 nuclear localization and toward testing whether enforcing membrane restriction versus nuclear enrichment of FXYD1 is sufficient to differentially modulate DDX5 stability and Wnt/β‐catenin transcriptional output.

A previous study by Jin et al. [23]. reported that FXYD1 is significantly downregulated in colorectal cancer tissues, with its expression negatively correlated with promoter methylation, suggesting that hypermethylation may mediate transcriptional silencing of FXYD1. Consistent with this, our findings demonstrate that promoter hypermethylation contributes to FXYD1 repression in breast cancer, supporting a conserved epigenetic mechanism of FXYD1 silencing across multiple cancer types. However, the upstream signals that initiate and maintain FXYD1 promoter methylation remain undefined in our current study. Previous reports indicate that oncogenic signaling pathways, such as PI3K/AKT activation or inflammatory cytokine signaling, can recruit DNA methyltransferases to tumor suppressor promoters [42, 43]. Whether similar mechanisms contribute to FXYD1 silencing remains to be determined and represents a limitation of our work. Future studies will aim to identify the epigenetic regulators responsible for FXYD1 methylation, which may offer new therapeutic opportunities to reactivate its expression in tumors.

It is well established that K48‐linked polyubiquitination serves as the canonical signal for proteasomal degradation, whereas K63‐linked chains are typically associated with non‐proteolytic functions, including signal transduction, endocytosis, and DNA damage responses [44]. Nevertheless, increasing evidence indicates that K63‐linked ubiquitin architectures can, under specific contexts, also be decoded by the proteasome in a topology‐ and receptor‐dependent manner [45, 46]. In yeast, purified 26S proteasomes can efficiently degrade substrates modified with homogeneous K63‐linked chains, and this process relies on intrinsic ubiquitin receptors such as Rpn10 [47]. Moreover, proteasomal receptors and shuttle factors display strong affinity for long K63‐linked chains (typically >7 ubiquitins), suggesting that chain length and receptor engagement can be a critical determinant for K63‐driven proteasomal targeting [47].

More recently, K63 ubiquitination has also been proposed to promote proteasome‐mediated degradation by serving as a “seed” for subsequent chain editing and the assembly of branched ubiquitin architectures that incorporate degradative linkages and preferentially engage proteasomes in cells [48, 49]. Importantly, our study did not directly resolve the full topology of DDX5‐linked ubiquitin chains, and we therefore do not infer the presence of branched K48/K63 chains on DDX5 from our data. Instead, our data support a proteasome‐dependent route for DDX5 turnover downstream of FXYD1: FXYD1‐induced DDX5 loss was rescued by MG132 but not by CQ, and DDX5 ubiquitination was abolished by the K63R (but not K48R) ubiquitin mutant, indicating a strict requirement for K63 linkage formation. Based on our finding that FXYD1 functions as a scaffold to promote MAEA‐dependent ubiquitination of DDX5, we propose that the FXYD1–MAEA axis may generate a K63‐dominant ubiquitin signal on DDX5 that is sufficient to trigger proteasomal engagement, potentially by promoting the assembly of longer K63‐linked chains that can be recognized by proteasomal ubiquitin receptors or shuttle proteins. These data extend the functional context in which K63‐linked ubiquitination can be coupled to proteasomal degradation and illustrate how scaffold proteins can shape ubiquitin signaling outcomes and substrate fate.

Moreover, we demonstrate that FXYD1 suppresses Wnt/β‐catenin signaling by promoting proteasomal degradation of DDX5, an oncogenic RNA helicase. However, the precise role of DDX5 in activating Wnt signaling is not fully characterized in our study. Accumulating evidence suggests that DDX5 plays a dual role in modulating β‐catenin activity. As a transcriptional coactivator, DDX5 interacts with β‐catenin to facilitate its recruitment to the promoters of Wnt target genes, thereby promoting transcriptional complex assembly and enhancing β‐catenin‐dependent transcription. Additionally, recent studies suggest that DDX5 may also contribute to β‐catenin protein stabilization by interfering with its phosphorylation‐dependent degradation, resulting in increased cytoplasmic accumulation and nuclear translocation [27, 33, 34, 50]. In line with these findings, our study shows that FXYD1‐mediated DDX5 degradation leads to a marked reduction in total β‐catenin protein levels and nuclear localization. These results support a model in which DDX5 enhances β‐catenin stability in the cytoplasm and promotes its nuclear import, collectively amplifying Wnt signaling output. By targeting DDX5 for degradation, FXYD1 simultaneously impairs β‐catenin stabilization and transcriptional activity, thereby inhibiting Wnt‐driven tumor progression. This highlights a multifaceted mechanism by which FXYD1 exerts its tumor‐suppressive effects and identifies the FXYD1–DDX5 axis as a promising therapeutic vulnerability in breast cancer.

As a limitation, our study was not originally designed to interrogate subtype‐specific biology of FXYD1, and the experimental work primarily relied on Luminal‐ and TNBC‐derived models. To address this concern, we performed subtype‐stratified analyses using complementary datasets: in the TCGA cohort, FXYD1 was consistently downregulated in tumors relative to normal breast tissues across the major intrinsic subtypes, with broadly comparable expression levels among Luminal, HER2‐positive, and TNBC tumors (Figure S9A); moreover, promoter methylation analyses in the same cohort indicated that FXYD1 exhibits a hypermethylated state in tumors across these subtypes (Figure S9B). In our institutional cohort, higher FXYD1 expression was associated with improved RFS within each subtype (Figure S9C–F). Together, these results support a pan‐subtype clinical relevance of FXYD1. Nevertheless, larger subtype‐resolved cohorts and additional representative models will be required to more precisely quantify subtype‐specific effect sizes.

Although our study provides strong preclinical evidence supporting the tumor‐suppressive function of FXYD1, we have not yet conducted translational investigations to evaluate its therapeutic potential. Based on our findings, two strategies could be envisioned for clinical application. First, reactivating FXYD1 expression in tumors via epigenetic therapies or CRISPR‐based gene reactivation could restore its tumor‐suppressive function and attenuate Wnt‐driven oncogenesis. Second, targeting the DDX5–MAEA interaction or developing molecules that mimic the scaffolding function of FXYD1 may offer an alternative approach to selectively degrade DDX5 and suppress Wnt signaling. However, these strategies merit further exploration using medicinal chemistry, high‐throughput screening, and patient‐derived models to evaluate their feasibility, efficacy, and specificity.

In conclusion, our study identifies FXYD1 as a novel tumor suppressor that modulates Wnt/β‐catenin signaling via a non‐canonical ubiquitin‐proteasome pathway. Further investigation into its upstream regulation, downstream functional consequences, and clinical applicability may yield important insights and offer new avenues for the development of targeted therapies in breast cancer.

## 4 Methods

### 4.1 Study Approval

Breast cancer tissue specimens were collected from Chongqing University Cancer Hospital with written informed consent obtained from all participants. The study protocol for the use of human tissues was reviewed and approved by the Institutional Ethics Committee of Chongqing University Cancer Hospital. All animal experiments were performed in strict accordance with the guidelines of the Institutional Animal Care and Treatment Committee of Chongqing University Cancer Hospital and complied with relevant institutional and national regulations (Approval No. CQCH‐LAE‐20231020025). The tumor burden in mice was strictly controlled and maintained below the maximum permissible limit (tumor diameter ≤ 1.5 cm).

### 4.2 Cell Culture

MDA‐MB‐231, BT‐549, T47D, SK‐BR‐3, BT‐474, MDA‐MB‐468, MCF‐7, MCF‐10A and HEK293T cells were obtained from the American Type Culture Collection (ATCC), as detailed in Table S4. MDA‐MB‐231, BT‐549, T47D, SK‐BR‐3 and BT‐474 cells were cultured in RPMI‐1640 with 10% fetal bovine serum (FBS; Gibco) and 1% penicillin‐streptomycin. MDA‐MB‐468, MCF‐7, and HEK293T cells were maintained in DMEM supplemented with 10% FBS. MCF‐10A cells were cultured in DMEM/F12 medium containing 5% horse serum, 10 mg/mL insulin, 0.5 mg/mL hydrocortisone, 20 ng/mL EGF, and 1% penicillin‐streptomycin. All cells were cultured at 37°C in a humidified incubator containing 5% CO_2_, authenticated by short tandem repeat profiling, and confirmed to be free of contamination and mycoplasma.

### 4.3 Reagents and Antibodies

MG132 (HY‐13259), Chloroquine (CQ; HY‐17589), and Cycloheximide (CHX; HY‐12320) were obtained from MedChemExpress. Lipofectamine 3000 (L3000015) was purchased from Thermo Fisher Scientific. The antibodies used for immunoblotting, immunofluorescence, immunohistochemical staining, and immunoprecipitation assays are provided in Table S5.

siRNA sequences (synthesized by Tsingke, China) are listed as follows:

si‐FXYD1 sense strand: 5’‐ACCCGUUCACUUACGACUA‐3’
si‐FXYD1 antisense strand: 5’‐UAGUCGUAAGUGAACGGGU‐3’

### 4.4 SMART‐Based DNA Methylation Analysis

DNA methylation was analyzed using the SMART App (Shiny Methylation Analysis Resource Tool), a Shiny‐based web application that queries TCGA datasets from the UCSC Xena public data hubs [51]. DNA methylation profiles generated on the Illumina HumanMethylation450K array were retrieved through SMART, with methylation levels reported as β values (0–1). CpG‐aggregated methylation was assessed using the platform's promoter/TSS‐associated CpG probe annotations. Tumor–normal comparisons were performed using the default statistical procedures implemented in SMART.

### 4.5 DNA Isolation and Bisulfite Sequencing PCR (BSP)

For DNA isolation, genomic DNA was isolated from cells and tissues utilizing a genomic DNA isolation kit (Thermo Fisher Scientific, K0512).

For BSP, it was performed and analyzed by SEQHEALTH biotech company (China). The methylation status was determined by cytosine retention (methylated) versus cytosine‐to‐thymine conversion (non‐methylated).

The primers used for BSP are listed as follows:

Human FXYD1 BSP Forward: 5’‐TAGGTTGTTGTTATGGTGGTTTGAG‐3’
Human FXYD1 BSP Reverse: 5’‐CAATATCACCAAAACAACCTCCAC‐3’

### 4.6 DNA Constructs and Mutagenesis

Human FXYD1 cDNA amplified by PCR was cloned into the pLV3‐CMV‐FLAG or pCMV‐FLAG‐EGFP‐BSD vector. Human CTNNB1 (β‐catenin) cDNA was inserted into the pLV3‐CMV‐Neo vector, and human DDX5 cDNA was cloned into either the pLV3‐CMV or pCMV‐3×HA‐Neo vector. Human MAEA cDNA was cloned into the pCMV‐6×His‐Neo vector. Point mutations of DDX5 (K207R, K236R, K343R, K375R, K437R, and K470R), ubiquitin (K48R, K63R, and K63‐only), and MAEA‐Y394A were generated and obtained from MiaoLing Plasmid (China).

### 4.7 Generation of Stable Cells

For gene silencing, single‐stranded shRNA was annealed and then ligated to the pLV‐H1‐EF1𝛼‐puro Vector (Biosettia, USA) according to the manufacturer's protocols. Lentiviral particles were produced using a three‐plasmid packaging system, and target cells were transduced and selected with puromycin (1 µg/mL) for 1 week.

The shRNA sequences are as follows:

shMAEA: 5’‐AAAAGCTTTCTATCCGTCAAGATTTGGATCCAAATCTTGACGGATAGAAAGC‐3’

For gene expression, pLV3‐CMV vector was used to stably express FXYD1, DDX5 and CTNNB1 in breast cancer cells. The lentivirus was produced using a three‐plasmid packaging system and transfected in cells, followed by selection with blasticidin or G418 (50 µg/mL) for 1 week.

### 4.8 Immunoblotting

Immunoblotting analysis was performed as previously described [52]. In brief, protein lysates from cultured cells were lysed with cold RIPA lysis containing protease and phosphatase inhibitor cocktail (Thermo Fisher Scientific, A32961). Protein concentration was determined by BCA assay (Beyotime, P0012). Equal amounts of protein were separated by SDS‐PAGE, transferred to PVDF membranes, and incubated with primary and HRP‐conjugated secondary antibodies. The blots were visualized by ECL assay, and the images were captured using a ChemiDoc MP system (Bio‐RAD, USA).

### 4.9 Immunoprecipitation and Mass Spectrometry (MS)

For immunoprecipitation, cells were lysed in cold RIPA lysis containing protease and phosphatase inhibitor cocktail (Thermo Fisher Scientific, A32961). Protein extracts were incubated with the specified primary antibody overnight at 4°C with gentle rotation. The following day, Protein A/G PLUS‐Agarose (Santa Cruz, sc‐2003) were added to each sample and incubated for 3 h at 4°C. The beads were then washed with RIPA lysis and analyzed by immunoblotting protocol.

For proteomic analysis, immunoprecipitates were resolved by SDS‐PAGE and stained with Coomassie Brilliant Blue (Beyotime, P0017A). Excised bands were processed and analyzed by MS at GeneCreat biotech company (China).

### 4.10 Immunofluorescence (IF) Staining

Cells were seeded on glass coverslips in a 24‐well plate and cultured for 24–48 h. After washing with PBS, cells were fixed with 4% paraformaldehyde for 10 min and permeabilized with 0.5% Triton X‐100 for 5 min, and washed three times with PBS containing 0.1% Tween‐20 (PBST). Samples were blocked with 3% BSA in PBST for 1 h, incubated with indicated primary antibody overnight at 4°C. Cells were then washed 3 times with 0.1% PBST and incubated with indicated secondary antibodies in 3% BSA for 1 h at room temperature in the dark. Following three additional PBST washes, coverslips were mounted with DAPI‐containing antifade medium and sealed with clear nail polish. Images were captured using a Leica STELLARIS 5 confocal microscope.

### 4.11 Immunohistochemical (IHC) Staining

The IHC staining was performed as previously described [52]. Briefly, the paraffin‐embedded slides were deparaffinized, rehydrated, and endogenous peroxidase activity blocked with 3% H_2_O_2_. Antigen retrieval was then conducted using a citrate buffer. The sections were then blocked with 10% goat serum at 37°C for 1 h, then incubated with primary antibodies overnight at 4°C. After washing, sections were incubated with appropriate secondary antibodies, developed with DAB, counterstained with hematoxylin, dehydrated, and mounted. IHC signals were semi‐quantitatively scored based on staining intensity (0, negative; 1, weak; 2, moderate; 3, strong) and the proportion of positively stained cells (0, 0%; 1, 1%–25%; 2, 26%–50%; 3, 51%–75%; 4, 76%–100%). A final IHC score was calculated by multiplying the intensity score by the proportion score, and the resulting values were used for statistical analyses. The staining intensity and percentage score were evaluated independently by two investigators.

### 4.12 Quantitative Real‐Time PCR (qRT‐PCR) Analysis

qRT‐PCR was performed as previously described [53]. Briefly, total RNA was extracted using TRIzol reagent (Thermo Fisher Scientific, 15596026) according to the manufacturer's instructions. Equal amounts of RNA were reverse‐transcribed into cDNA using a commercially available reverse transcription kit (Yeasen, 11137ES) following the supplier's protocol. qRT‐PCR was performed using SYBR Green Master Mix (Bimake, B21203) according to manufacturer's protocol. Relative gene expression was normalized to glyceraldehyde‐3‐phosphate dehydrogenase (GAPDH) and calculated using the 2‐ΔΔCt method. Primer sequences are provided in Table S6.

### 4.13 Proliferation Assay

For the CCK‐8 assay, cells were seeded in 96‐well plates at 2 ×  10^3^ cells per well. After attachment, the culture medium was replaced with CCK‐8 reagent according to the manufacturer's instructions. Plates were incubated in the dark at 37°C for 2 h, and absorbance was measured at 450 nm using a microplate reader (Bio‐RAD, USA).

For the colony formation assay, cells were seeded in 6‐well plates at 5 × 10^2^ cells per well and cultured until visible colonies formed. Then, the colonies were fixed with 4% paraformaldehyde and stained with 0.1% crystal violet, followed by image acquisition and count.

### 4.14 Wound‐Healing Assay

Cells were seeded in 6‐well plates and grown to 90%–95% confluence. A straight scratch was made across the monolayer using a sterile 10 µL pipette tip. After gently washing with PBS to remove detached cells, cultures were maintained in serum‐free medium. Images were captured at an indicated time point by a microscope (Olympus, Japan).

### 4.15 Migration and Invasion Assays

Breast cancer cells were resuspended in 200 µL serum‐free medium and seeded into 8 µm‐pore transwell inserts (BD Biosciences, USA), which were pre‐coated with Matrigel for invasion assays or left uncoated for migration assays. Inserts were placed in 24‐well plates containing 500 µL complete medium and incubated for the indicated time. After incubation, cells on the lower membrane surface were fixed in 4% paraformaldehyde for 15 min at room temperature, stained with 0.1% crystal violet for 30 min, and non‐invaded/non‐migrated cells on the upper surface were gently removed with a cotton swab. Images were acquired using an optical microscope (Olympus, Japan). The number of migrated or invaded cells were counted in five random fields per replicate.

### 4.16 Invadopodia Formation Assay

Cells were fixed, permeabilized, and incubated with an anti‐cortactin primary antibody followed by indicated fluorescent secondary antibody, then stained with phalloidin to visualize F‐actin. Invadopodia were identified by cortactin and F‐actin co‐localization. Five random fields per sample were imaged and quantified.

### 4.17 GST Pulldown Assay

For pulldown assays, recombinant human GST‐DDX5 or GST protein was incubated with His‐FXYD1, GST‐DDX5 or GST protein with His‐MAEA, and GST‐MAEA or GST protein with His‐FXYD1 in NP‐40 buffer at 4°C overnight. The mixtures were subsequently incubated with GST beads (Smart Lifesciences, SA008005) for 3 h at 4°C. After extensive washing with NP‐40 buffer, bound proteins were eluted by boiling in SDS loading buffer and analyzed by immunoblotting.

### 4.18 Animal Studies

BALB/c nude mice (5‐week‐old female, GemPharmatech) were housed under ambient temperature of 24 ± 2°C, circulating air, constant humidity of 50% ± 10%, and a 12 h:12 h light/dark cycle. For subcutaneous xenografts, 2 × 10^6^ MDA‐MB‐231 cells were suspended in PBS and inoculated into the flank of nude mice. Tumor volume was measured every three days (volume = length × width^2^ × 0.5). For the lung metastasis assays, 1 × 10^6^ cells in 100 µL PBS were injected via the tail‐vein. At the study endpoint, mice were euthanized, and tumors and lungs were collected for analysis. Each group comprised five mice (n = 5). All procedures were approved by the Ethics Committee of Chongqing University Cancer Hospital.

### 4.19 Statistical Analysis

Statistical analyses were performed using GraphPad Prism 10 software. Statistical significance was determined by one‐sided unpaired Student's test between two groups, or one‐way ANOVA for comparisons among multiple groups. Pearson correlation and linear regression were used to determine the correlation in clinical samples. Methods for statistical tests and exact value of n were indicated in figure legends. All data are presented as mean ± SD and represent at least three independent experiments. Differences were considered statistically significant at *p* < 0.05.

## Author Contributions

Ningning Zhang, Guanwen Wang, Ping Wen, Jiangdong Sui, and Xiaohua Zeng conceived and designed the study. Ping Wen, Fanli Qu, Qing Shao, Sisi Li, and Yang Qin performed the experiments. Ping Wen, Guanwen Wang, Fanli Qu, Qing Shao, Long Wang, Dongping Jiang, Senmiao Zhang, Ningning Zhang, and Xiaohua Zeng analyzed and interpreted the data. Ping Wen, Guanwen Wang, Ningning Zhang, Jiangdong Sui, and Xiaohua Zeng drafted and revised the manuscript. All authors read and approved the final version of the manuscript.

## Funding

This study was supported by National Natural Science Foundation of China (Grant Nos. 82573237, 82302960 and 82303618), Natural Science Foundation of Chongqing (Grant Nos. CSTB2024NSCQ‐MSX0694, CSTB2024NSCQ‐MSX1087 and CSTB2025NSCQ‐GPX0696), Chongqing Science and Health Joint Medical Research Project (Grant Nos. 2025ZDXM029 and 2025MSXM005), Special Key Projects of Technological Innovation and Application Development in Chongqing (Grant No. CSTB2023TIAD‐KPX0049‐5), Chongqing Medical Youth Top Talent Program (Grant No. YXQN202475), Science and Technology Research Program of Chongqing Municipal Education Commission (Grant Nos. KJQN202400134 and KJQN202500129).

## Conflicts of Interest

The authors declare no conflicts of interest.

## Ethics Approval and Consent to Participate

The clinical samples and information used in the study were approved by the Institutional Ethics Committee of Chongqing University Cancer Hospital. The animal experiments were conducted in compliance with protocols approved by the Institutional Animal Care and Treatment Committee of Chongqing University Cancer Hospital.

## Supporting information




**Supporting File**: advs74694‐sup‐0001‐SuppMat.pdf

## Data Availability

The data that support the findings of this study are available from the corresponding author upon reasonable request.

## References

[1] F. Bray , M. Laversanne , H. Sung , et al., “Global Cancer Statistics 2022: GLOBOCAN Estimates of Incidence and Mortality Worldwide for 36 Cancers in 185 Countries,” CA: A Cancer Journal for Clinicians 74, no. 3 (2024): 229–263, 10.3322/caac.21834. 38572751

[2] X. Xiong , L.‐W. Zheng , Y. Ding , et al., “Breast Cancer: Pathogenesis and Treatments,” Signal Transduction and Targeted Therapy 10, no. 1 (2025): 49, 10.1038/s41392-024-02108-4. 39966355 PMC11836418

[3] Y. Zhang , Y. Tan , J. Yuan , et al., “circLIFR‐007 Reduces Liver Metastasis via Promoting hnRNPA1 Nuclear Export and YAP Phosphorylation in Breast Cancer,” Cancer Letters 592 (2024): 216907, 10.1016/j.canlet.2024.216907. 38685451

[4] K. Ganesan , C. Xu , S. Wu , et al., “Ononin Inhibits Tumor Bone Metastasis and Osteoclastogenesis by Targeting Mitogen‐Activated Protein Kinase Pathway in Breast Cancer,” Research 7 (2024): 0553, 10.34133/research.0553. 39687715 PMC11648741

[5] J. Xie , W. Liu , X. Deng , et al., “Paracrine Orchestration of Tumor Microenvironment Remodeling Induced by GLO1 Potentiates Lymph Node Metastasis in Breast Cancer,” Advanced Science 12, no. 32 (2025): 00722, 10.1002/advs.202500722. PMC12407353 40492378

[6] Y. Xie , J. Xie , G. Huang , et al., “Isoliquiritigenin Reduces Brain Metastasis by circNAV3‐ST6GALNAC5‐EGFR Axis in Triple‐Negative Breast Cancer,” Cancer Letters 624 (2025): 217734, 10.1016/j.canlet.2025.217734. 40268132

[7] T. Fleischer , X. Tekpli , A. Mathelier , et al., “DNA Methylation at Enhancers Identifies Distinct Breast Cancer Lineages,” Nature Communications 8, no. 1 (2017): 1379, 10.1038/s41467-017-00510-x. PMC5680222 29123100

[8] B. Pasculli , R. Barbano , and P. Parrella , “Epigenetics of Breast Cancer: Biology and Clinical Implication in the Era of Precision Medicine,” Seminars in Cancer Biology 51 (2018): 22–35, 10.1016/j.semcancer.2018.01.007. 29339244

[9] X. Ou , Y. Tan , J. Xie , et al., “Methylation of GPRC5A Promotes Liver Metastasis and Docetaxel Resistance Through Activating mTOR Signaling Pathway in Triple Negative Breast Cancer,” Drug Resistance Updates 73 (2024): 101063, 10.1016/j.drup.2024.101063. 38335844

[10] J. Zhang , Z. Huang , C. Song , et al., “PRMT1‐Mediated PARP1 Methylation Drives Lung Metastasis and Chemoresistance via P65 Activation in Triple‐Negative Breast Cancer,” Research 8 (2025): 0854, 10.34133/research.0854. 40927753 PMC12415337

[11] A. V. Lee , K. A. Nestler , and K. B. Chiappinelli , “Therapeutic Targeting of DNA Methylation Alterations in Cancer,” Pharmacology & Therapeutics 258 (2024): 108640, 10.1016/j.pharmthera.2024.108640. 38570075

[12] H. Ozturk , H. Cingoz , T. Tufan , et al., “ISL2 is a Putative Tumor Suppressor whose Epigenetic Silencing Reprograms the Metabolism of Pancreatic Cancer,” Developmental Cell 57, no. 11 (2022): 1331–1346.e9, 10.1016/j.devcel.2022.04.014. 35508175

[13] Z. Yuan , X. Yu , W. Chen , et al., “Epigenetic Silencing and Tumor Suppressor Gene of HAND2 by Targeting ERK Signaling in Colorectal Cancer,” Cell Communication and Signaling 20, no. 1 (2022): 111, 10.1186/s12964-022-00878-4. 35870943 PMC9308366

[14] A. Litan and S. A. Langhans , “Cancer as a Channelopathy: Ion Channels and Pumps in Tumor Development and Progression,” Frontiers in Cellular Neuroscience 9, (2015): 86, 10.3389/fncel.2015.00086. 25852478 PMC4362317

[15] G. Fnu and G. F. Weber , “Alterations of Ion Homeostasis in Cancer Metastasis: Implications for Treatment,” Frontiers in Oncology 11 (2021): 765329, 10.3389/fonc.2021.765329. 34988012 PMC8721045

[16] J. Q. Yap , J. Seflova , R. Sweazey , P. Artigas , and S. L. Robia , “FXYD Proteins and Sodium Pump Regulatory Mechanisms,” Journal of General Physiology 153, no. 4 (2021), 10.1085/jgp.202012633. PMC7953255 33688925

[17] Y. Shimada , S. Yamasaki , Y. Hashimoto , et al., “Clinical Significance of Dysadherin Expression in Gastric Cancer Patients,” Clinical Cancer Research 10, no. 8 (2004): 2818–2823, 10.1158/1078-0432.ccr-0633-03. 15102690

[18] A. Batistatou , D. Peschos , H. Tsanou , et al., “In Breast Carcinoma Dysadherin Expression Is Correlated With Invasiveness but Not With E‐Cadherin,” British Journal of Cancer 96, no. 9 (2007): 1404–1408, 10.1038/sj.bjc.6603743. 17437014 PMC2360179

[19] Z.‐L. Zhu , Z.‐R. Zhao , Y. Zhang , et al., “Expression and Significance of FXYD‐3 Protein in Gastric Adenocarcinoma,” Disease Markers 28, no. 2 (2010): 63–69, 10.3233/DMA-2010-0669. 20364041 PMC3833338

[20] H. Kayed , J. Kleeff , A. Kolb , et al., “FXYD3 Is Overexpressed in Pancreatic Ductal Adenocarcinoma and Influences Pancreatic Cancer Cell Growth,” International Journal of Cancer 118, no. 1 (2006): 43–54, 10.1002/ijc.21257. 16003754

[21] Y. Xue , L. Lai , W. Lian , et al., “SOX9/FXYD3/Src Axis Is Critical for ER+ Breast Cancer Stem Cell Function,” Molecular Cancer Research 17, no. 1 (2019): 238–249, 10.1158/1541-7786.MCR-18-0610. 30206184

[22] E. Zhao , K. Gao , J. Xiong , Z. Liu , Y. Chen , and L. Yi , “The Roles of FXYD Family Members in Ovarian Cancer: An Integrated Analysis by Mining TCGA and GEO Databases and Functional Validations,” Journal of Cancer Research and Clinical Oncology 149, no. 19 (2023): 17269–17284, 10.1007/s00432-023-05445-z. 37814066 PMC11796877

[23] M. Jin , H. Zhang , J. Yang , Z. Zheng , and K. Liu , “Expression Mode and Prognostic Value of FXYD Family Members in Colon Cancer,” Aging 13, no. 14 (2021): 18404–18422, 10.18632/aging.203290. 34270462 PMC8351680

[24] X. P. Tan , B. H. Xiong , Y. X. Zhang , S. L. Wang , Q. Zuo , and J. Li , “FXYD5 promotes Sorafenib Resistance Through the Akt/mTOR Signaling Pathway in Hepatocellular Carcinoma,” European Journal of Pharmacology 931 (2022): 175186, 10.1016/j.ejphar.2022.175186. 35977595

[25] W. Yang , R. He , H. Qu , et al., “FXYD3 enhances IL‐17A Signaling to Promote Psoriasis by Competitively Binding TRAF3 in Keratinocytes,” Cellular & Molecular Immunology 20, no. 3 (2023): 292–304, 10.1038/s41423-023-00973-7. 36693922 PMC9971024

[26] X. Zhao , D. Li , F. Yang , et al., “Long Noncoding RNA NHEG1 Drives β‐Catenin Transactivation and Neuroblastoma Progression Through Interacting With DDX5,” Molecular Therapy 28, no. 3 (2020): 946–962, 10.1016/j.ymthe.2019.12.013. 31982037 PMC7054727

[27] S. Shin , K. L. Rossow , J. P. Grande , and R. Janknecht , “Involvement of RNA Helicases p68 and p72 in Colon Cancer,” Cancer Research 67, no. 16 (2007): 7572–7578, 10.1158/0008-5472.CAN-06-4652. 17699760

[28] M. Zhang , W. Weng , Q. Zhang , et al., “The lncRNA NEAT1 Activates Wnt/Beta‐catenin Signaling and Promotes Colorectal Cancer Progression via Interacting With DDX5,” Journal of Hematology & Oncology 11, no. 1 (2018): 113, 10.1186/s13045-018-0656-7. 30185232 PMC6125951

[29] E. S. Bell , P. Shah , N. Zuela‐Sopilniak , et al., “Low Lamin A Levels Enhance Confined Cell Migration and Metastatic Capacity in Breast Cancer,” Oncogene 41, no. 36 (2022): 4211–4230, 10.1038/s41388-022-02420-9. 35896617 PMC9925375

[30] M. Sgubin , S. Pegoraro , I. Pellarin , et al., “HMGA1 positively Regulates the Microtubule‐Destabilizing Protein Stathmin Promoting Motility in TNBC Cells and Decreasing Tumour Sensitivity to Paclitaxel,” Cell Death & Disease 13, no. 5 (2022): 429, 10.1038/s41419-022-04843-4. 35504904 PMC9065117

[31] W. H. Yang , M. J. Ding , G. Z. Cui , M. Yang , and D. L. Dai , “Heterogeneous Nuclear ribonucleoprotein M Promotes the Progression of Breast Cancer by Regulating the Axin/Beta‐catenin Signaling Pathway,” Biomedicine & Pharmacotherapy 105 (2018): 848–855, 10.1016/j.biopha.2018.05.014. 30021377

[32] K. Li , G. Zhao , H. Yuan , et al., “Upregulated Expression of DDX5 Predicts Recurrence and Poor Prognosis in Breast Cancer,” Pathology—Research and Practice 229 (2022): 153736, 10.1016/j.prp.2021.153736. 34923193

[33] K. K. Guturi , M. Sarkar , A. Bhowmik , N. Das , and M. K. Ghosh , “DEAD‐Box Protein p68 Is Regulated by Beta‐Catenin/Transcription Factor 4 to Maintain a Positive Feedback Loop in Control of Breast Cancer Progression,” Breast Cancer Research 16, no. 6 (2014): 496, 10.1186/s13058-014-0496-5. 25499975 PMC4308923

[34] W. Tian , Y. Tang , Y. Luo , et al., “AURKAIP1 actuates Tumor Progression Through Stabilizing DDX5 in Triple Negative Breast Cancer,” Cell Death & Disease 14, no. 12 (2023): 790, 10.1038/s41419-023-06115-1. 38040691 PMC10692340

[35] S. Kolla , M. Ye , K. G. Mark , and M. Rape , “Assembly and Function of Branched Ubiquitin Chains,” Trends in Biochemical Sciences 47, no. 9 (2022): 759–771, 10.1016/j.tibs.2022.04.003. 35508449

[36] X. Li , K.‐B. Yang , W. Chen , et al., “CUL3 (cullin 3)‐Mediated Ubiquitination and Degradation of BECN1 (beclin 1) Inhibit Autophagy and Promote Tumor Progression,” Autophagy 17, no. 12 (2021): 4323–4340, 10.1080/15548627.2021.1912270. 33977871 PMC8726624

[37] Y. Qian , Z. Wang , H. Lin , et al., “TRIM47 Is a Novel Endothelial Activation Factor That Aggravates Lipopolysaccharide‐induced Acute Lung Injury in Mice via K63‐linked Ubiquitination of TRAF2,” Signal Transduction and Targeted Therapy 7, no. 1 (2022): 148, 10.1038/s41392-022-00953-9. 35513381 PMC9072678

[38] V. N. Jordan , A. Ordureau , and H. An , “Identifying E3 Ligase Substrates with Quantitative Degradation Proteomics,” Chembiochem 24, no. 16 (2023): 202300108, 10.1002/cbic.202300108. PMC10548883 37166757

[39] S. A. Yi , S. Sepic , B. A. Schulman , A. Ordureau , and H. An , “mTORC1‐CTLH E3 Ligase Regulates the Degradation of HMG‐CoA Synthase 1 Through the Pro/N‐degron Pathway,” Molecular Cell 84, no. 11 (2024): 2166–2184e9, 10.1016/j.molcel.2024.04.026. 38788716 PMC11186538

[40] S. Y. Lin , K. Makino , W. Xia , et al., “Nuclear Localization of EGF Receptor and Its Potential New Role as a Transcription Factor,” Nature Cell Biology 3, no. 9 (2001): 802–808, 10.1038/ncb0901-802. 11533659

[41] Y. Liu , P. Li , L. Fan , and M. Wu , “The Nuclear Transportation Routes of Membrane‐Bound Transcription Factors,” Cell Communication and Signaling 16, no. 1 (2018): 12, 10.1186/s12964-018-0224-3. 29615051 PMC5883603

[42] P. Liu , F. Yang , L. Zhang , et al., “Emerging Role of Different DNA Methyltransferases in the Pathogenesis of Cancer,” Frontiers in Pharmacology 13 (2022): 958146, 10.3389/fphar.2022.958146. 36091786 PMC9453300

[43] Z. Yu , J. Feng , W. Wang , et al., “The EGFR‐ZNF263 Signaling Axis Silences SIX3 in Glioblastoma Epigenetically,” Oncogene 39, no. 15 (2020): 3163–3178, 10.1038/s41388-020-1206-7. 32051553 PMC7142014

[44] F. Liu , J. Chen , K. Li , et al., “Ubiquitination and Deubiquitination in Cancer: From Mechanisms to Novel Therapeutic Approaches,” Molecular Cancer 23, no. 1 (2024): 148, 10.1186/s12943-024-02046-3. 39048965 PMC11270804

[45] L. Wu , X. Yan , R. Sun , et al., “Sirt3 Restricts Tumor Initiation via Promoting LONP1 Deacetylation and K63 Ubiquitination,” Journal of Translational Medicine 21, no. 1 (2023): 81, 10.1186/s12967-023-03925-x. 36739437 PMC9899405

[46] S. Qin , F. Chang , X. Sun , Z. Li , Y. Wang , and D. Lei , “TRIM47 Promotes Hypopharyngeal and Laryngeal Cancers Progression Through Promoting K63 ‐Linked Ubiquitination of VIMENTIN,” Cancer Science 116, no. 2 (2025): 367–380, 10.1111/cas.16397. 39584529 PMC11786321

[47] Y. Saeki , T. Kudo , T. Sone , et al., “Lysine 63‐linked Polyubiquitin Chain May Serve as a Targeting Signal for the 26S Proteasome,” The EMBO Journal 28, no. 4 (2009): 359–371, 10.1038/emboj.2008.305. 19153599 PMC2646160

[48] H. J. Meyer and M. Rape , “Enhanced Protein Degradation by Branched Ubiquitin Chains,” Cell 157, no. 4 (2014): 910–921, 10.1016/j.cell.2014.03.037. 24813613 PMC4028144

[49] F. Ohtake , H. Tsuchiya , Y. Saeki , and K. Tanaka , “K63 Ubiquitylation Triggers Proteasomal Degradation by Seeding Branched Ubiquitin Chains,” Proceedings of the National Academy of Sciences 115, no. 7 (2018): E1401–E1408, 10.1073/pnas.1716673115. PMC5816176 29378950

[50] Q. Fu , X. Song , Z. Liu , et al., “miRomics and Proteomics Reveal a miR‐296‐3p/PRKCA/FAK/Ras/c‐Myc Feedback Loop Modulated by HDGF/DDX5/β‐catenin Complex in Lung Adenocarcinoma,” Clinical Cancer Research 23, no. 20 (2017): 6336–6350, 10.1158/1078-0432.CCR-16-2813. 28751441

[51] Y. Li , D. Ge , and C. Lu , “The SMART App: An Interactive Web Application for Comprehensive DNA Methylation Analysis and Visualization,” Epigenetics & Chromatin 12, no. 1 (2019): 71, 10.1186/s13072-019-0316-3. 31805986 PMC6894252

[52] G. Wang , P. Wen , T. Xue , et al., “Her2 promotes Early Dissemination of Breast Cancer by Inhibiting the p38 Pathway Through the Downregulation of MAP3K4,” Cell Communication and Signaling 22, no. 1 (2024): 611, 10.1186/s12964-024-02000-2. 39702199 PMC11660853

[53] P. Wen , D. Jiang , F. Qu , et al., “PFDN5 plays a Dual Role in Breast Cancer and Regulates Tumor Immune Microenvironment: Insights From Integrated Bioinformatics Analysis and Experimental Validation,” Gene 933 (2025): 149000, 10.1016/j.gene.2024.149000. 39396557

