# Supplementary material for: Promoter Hypermethylation‐Induced Silencing of FXYD1 Drives Breast Cancer Metastasis via DDX5‐Mediated Wnt/β‐Catenin Pathway Activation
Source: Adv Sci (Weinh). 2026 Mar 4;13(27):e21921. doi: 10.1002/advs.202521921 (PMC13170254; doi:10.1002/advs.202521921)
Supplement: Supplementary file 1 — Supporting File: advs74694‐sup‐0001‐SuppMat.pdf [file ADVS-13-e21921-s001.pdf]

Supplementary Information

Promoter Hypermethylation-Induced Silencing of FXYD1 Drives Breast Cancer Metastasis via DDX5-Mediated Wnt/β-catenin Pathway Activation

Ping Wen, Fanli Qu, Long Wang, Qing Shao, Sisi Li, Yang Qin, Dongping Jiang,

Senmiao Zhang, Jiangdong Sui*, Guanwen Wang*, Ningning Zhang*, Xiaohua Zeng*

Contents

Supplementary Figures 1-9

Supplementary Figure Legends

Supplementary Tables 1-6

Supplementary Figures and legends

Figure S1

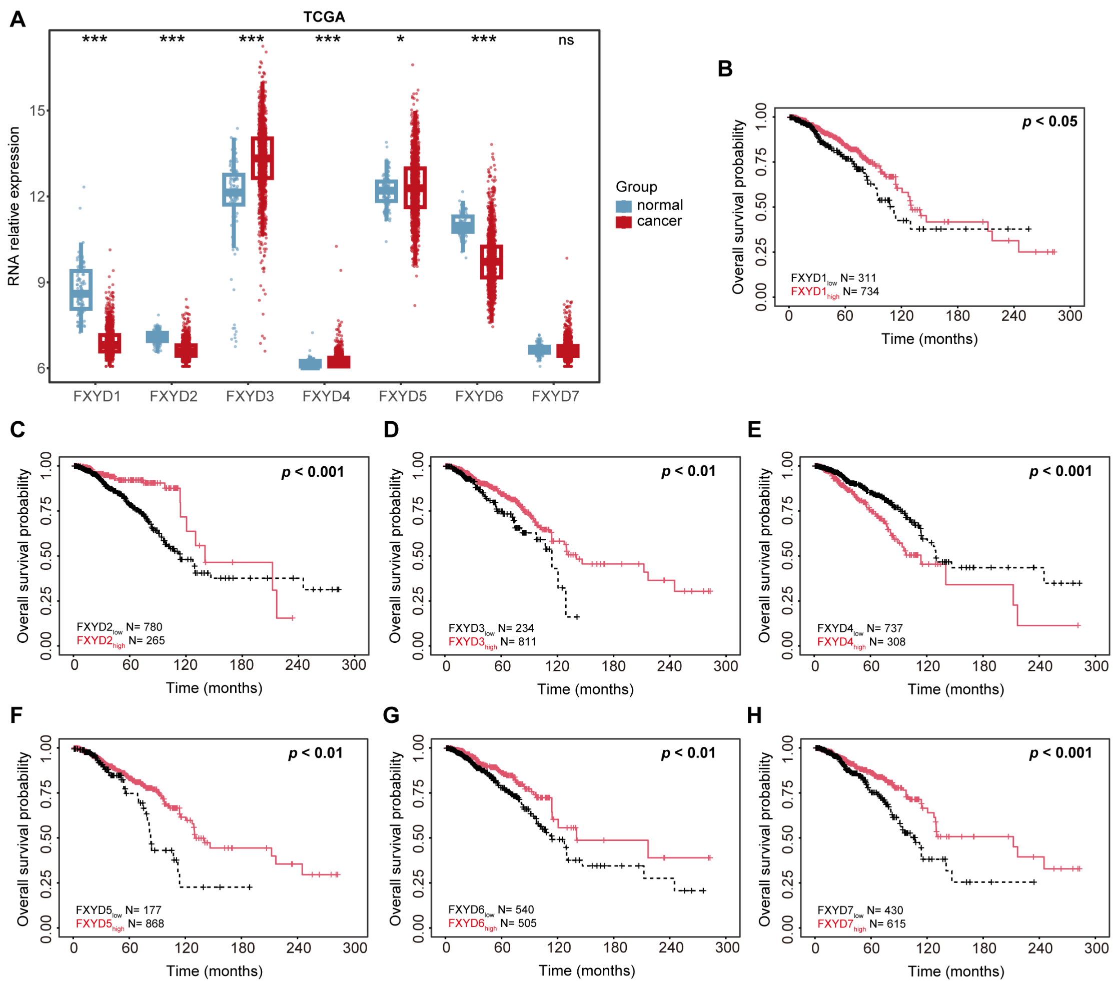

Supplementary Figure 1. Expression patterns and prognostic relevance of FXYD family genes in breast cancer based on the TCGA cohort. (A) mRNA expression profiles of FXYD family genes in breast cancer based on the TCGA cohort. (B–H) Kaplan–Meier survival analysis evaluating the correlation between the expression levels of individual FXYD family genes and OS in breast cancer patients based on the TCGA cohort. Patients were stratified into high- and low-expression groups based on the optimal cut-off value.

Figure S2

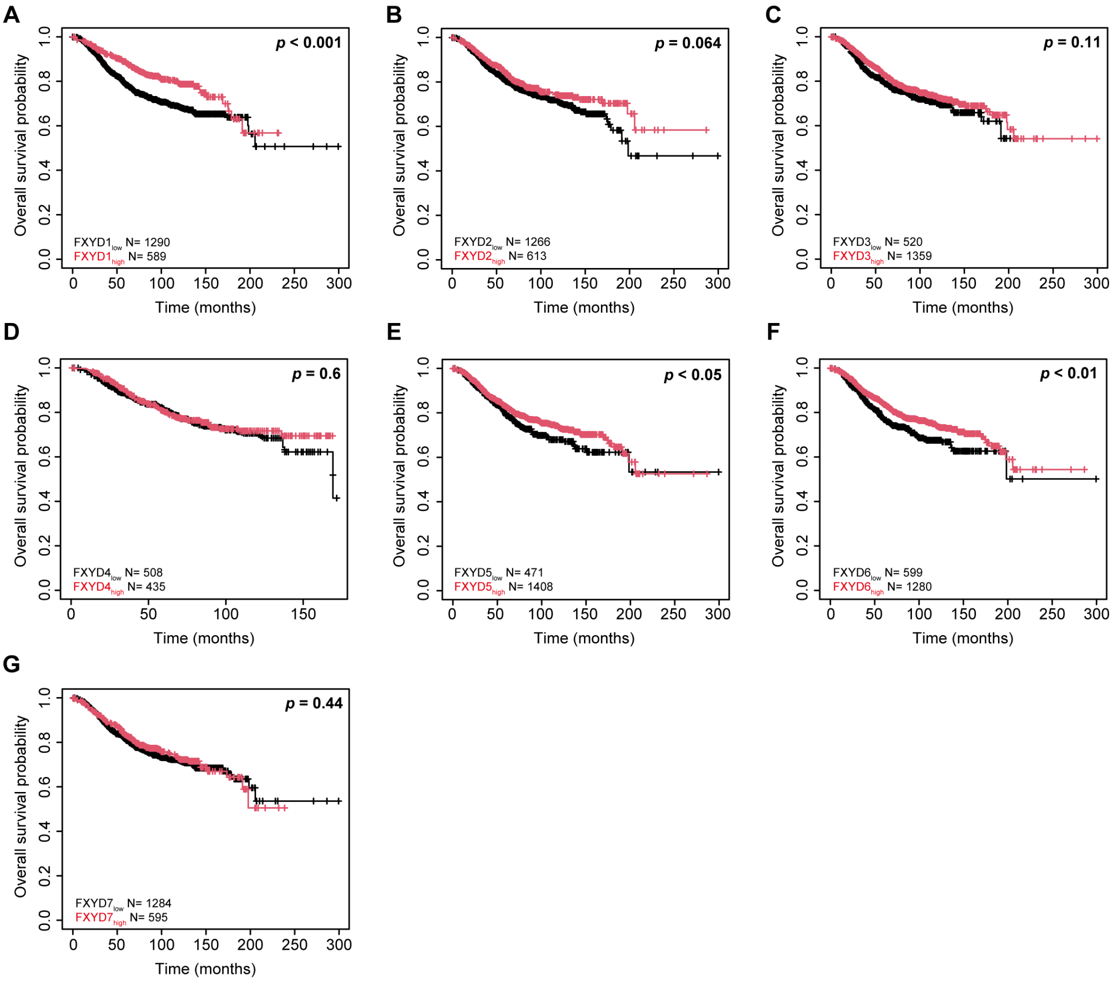

Supplementary Figure 2. Associations between FXYD family genes and OS in breast cancer based on the KM-plotter cohort. (A–G) Kaplan–Meier survival analysis evaluating the correlation between the expression levels of individual FXYD family genes and OS in breast cancer patients based on the KM-plotter cohort. Patients were stratified into high- and low-expression groups based on the optimal cut-off value.

Figure S3

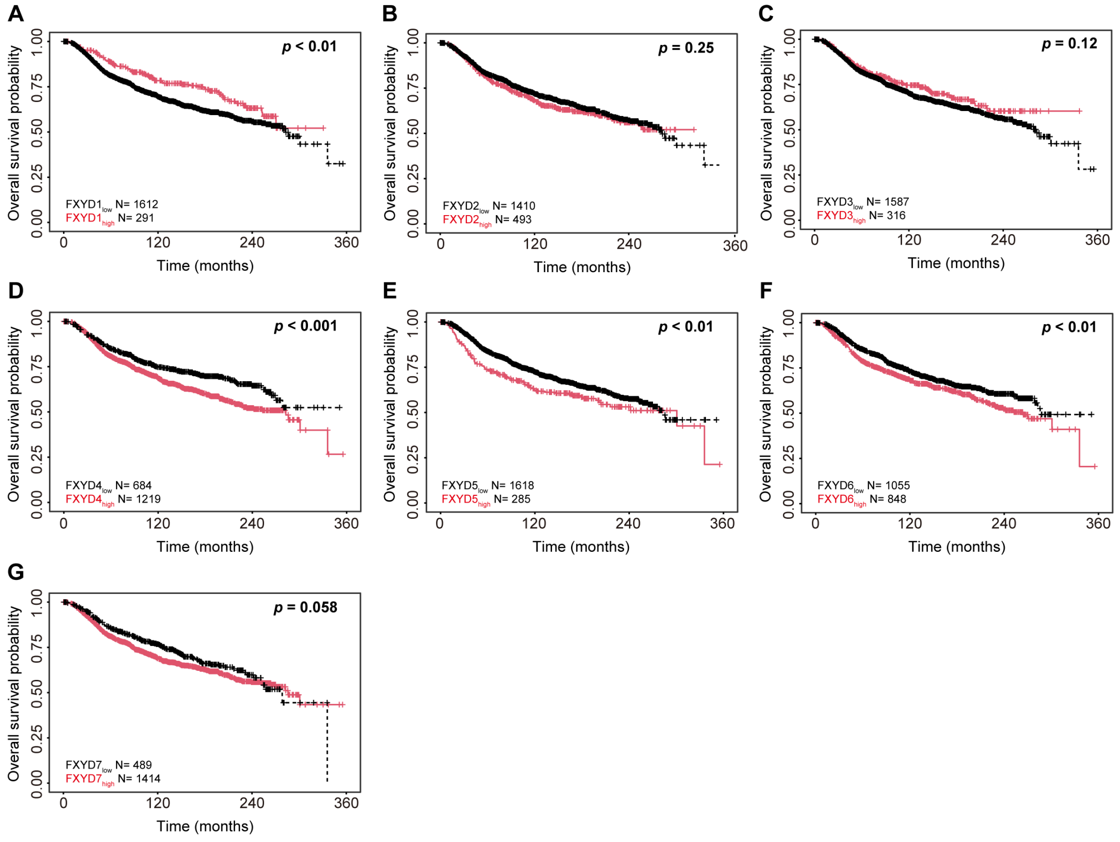

Supplementary Figure 3. Associations between FXYD family genes and OS in breast cancer based on the METABRIC cohort. (A–G) Kaplan–Meier survival analysis evaluating the correlation between the expression levels of individual FXYD family genes and OS in breast cancer patients based on the METABRIC cohort. Patients were stratified into high- and low-expression groups based on the optimal cut-off value.

Figure S4

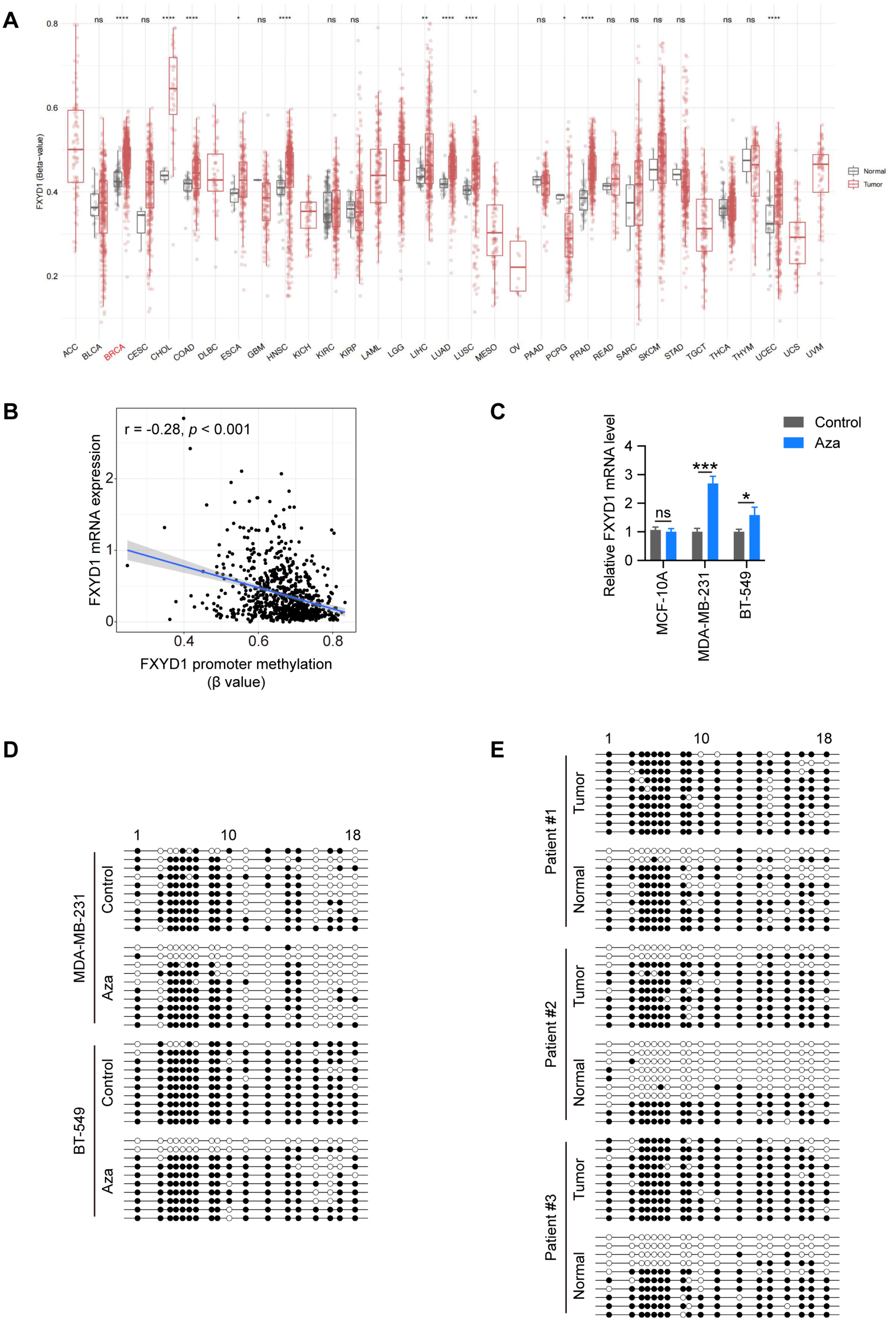

Supplementary Figure 4. Promoter hypermethylation contributes to FXYD1

transcriptional silencing in breast cancer. (A) Analysis of FXYD1 promoter methylation levels across multiple cancers using the SMART online tool. (B) Scatter plot showing the association between FXYD1 promoter methylation (β value) and FXYD1 mRNA expression in the TCGA cohort. (C) qRT-PCR analysis of FXYD1 expression in MCF-10A, MDA-MB-231, and BT-549 cells with or without Aza treatment (2 μM, 24 h). (D, E) BSP analysis of FXYD1 promoter methylation in MDA-MB-231 and BT-549 cells treated with or without Aza (2 μM, 24 h) (C), and in three paired breast cancer and adjacent normal tissues (D). ns, not significant, *$p < 0.05$, ***$p < 0.001$ vs. indicated controls in one-sided unpaired t-test.

Figure S5

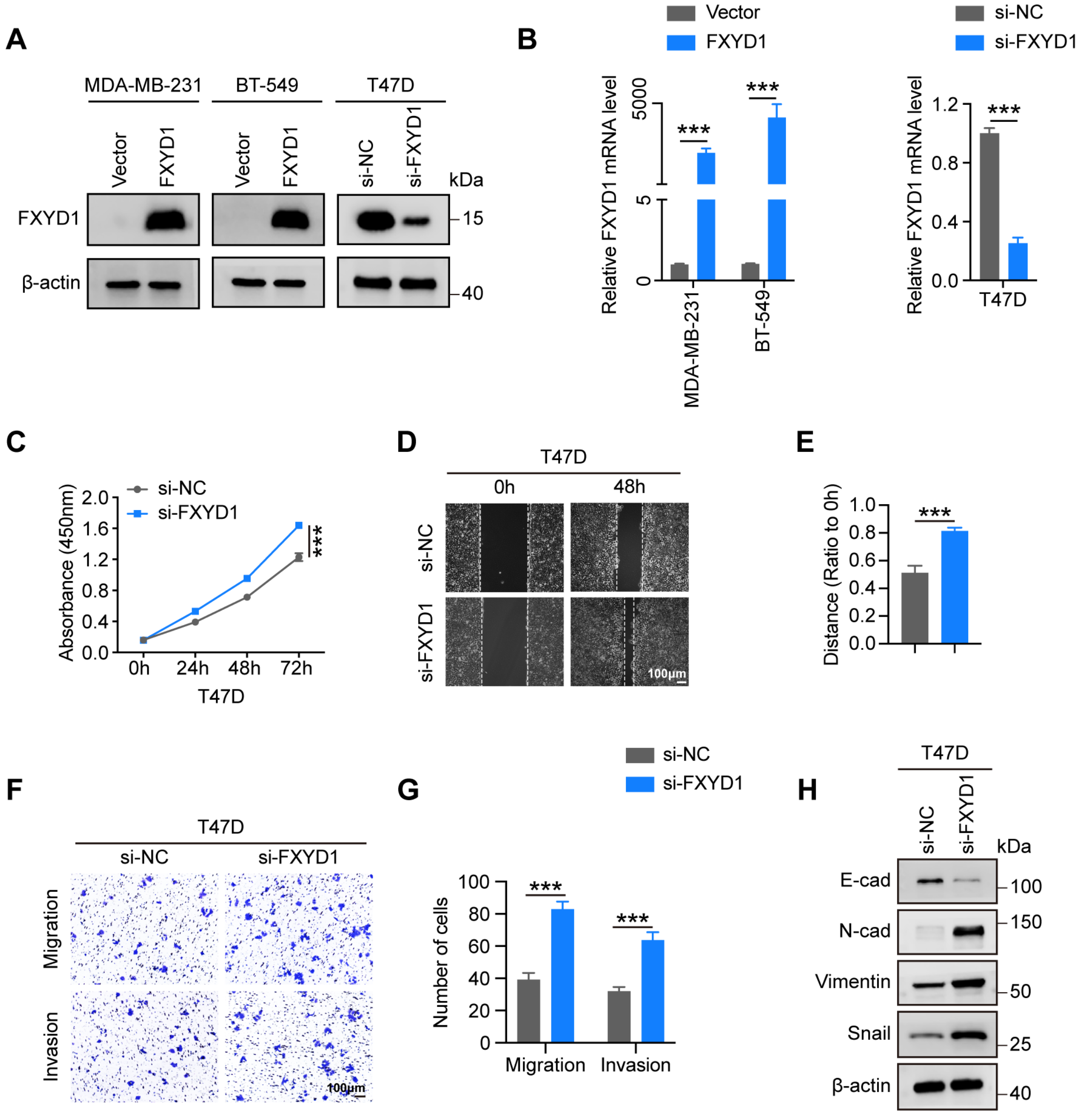

Supplementary Figure 5. FXYD1 knockdown promotes breast cancer cell proliferation and metastasis in vitro. (A, B) Immunoblot (A) and qRT-PCR (B) showing FXYD1 expression in FXYD1 overexpressing MDA-MB-231 and BT-549 cells, and FXYD1 knockdown T47D cells. (C) Cell proliferation was assessed by CCK-8 assay. (D, E) The wound-healing assays detecting the migration. (F, G) Transwell assays assessing cell migration and invasion capacities. (H) Immunoblot analysis of EMT-related markers, including E-cadherin, N-cadherin, Vimentin, and the EMT-associated transcription factor Snail. Data are presented as the mean ± SD (n = 3 independent

experiments). ***p < 0.001 vs. indicated controls in one-sided unpaired t-test.

Figure S6

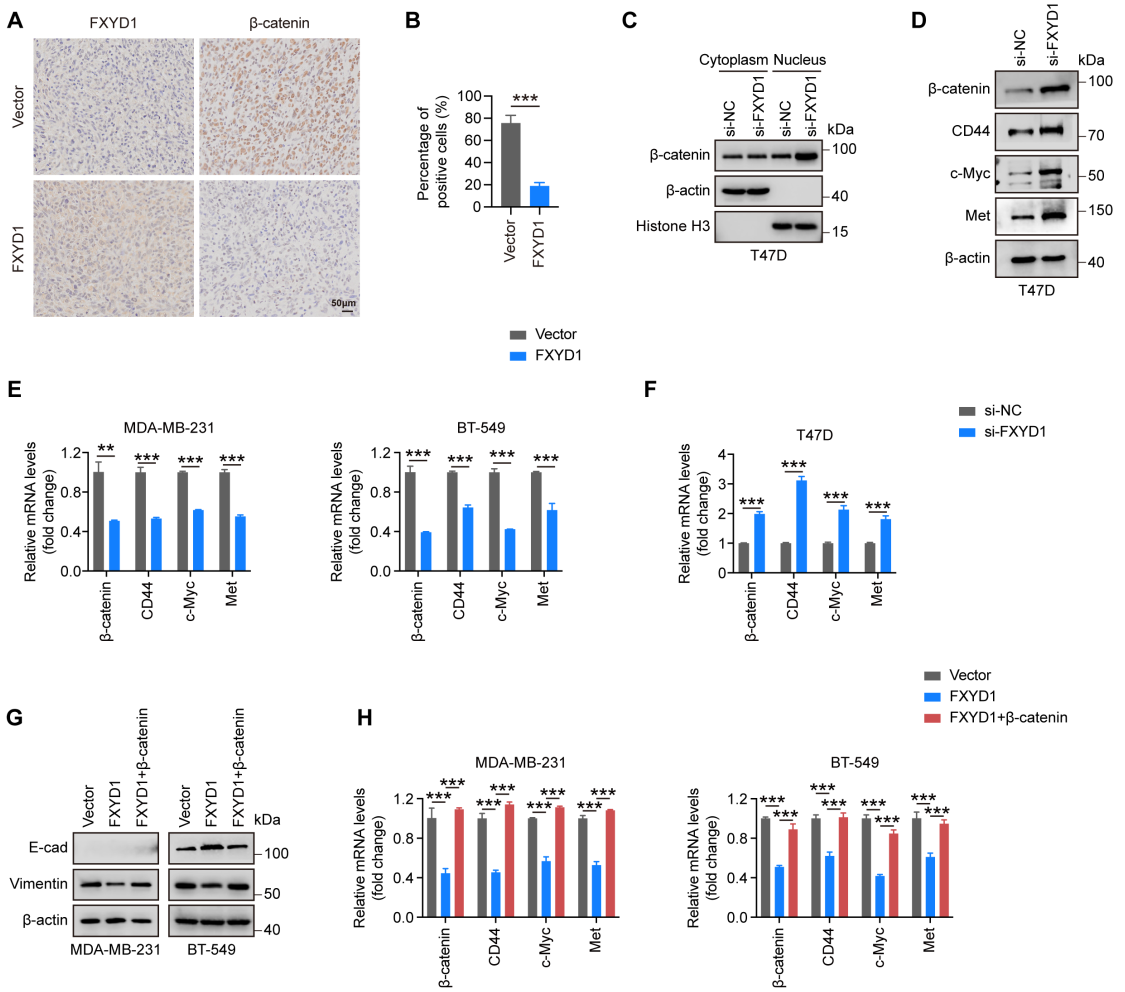

Supplementary Figure 6. FXYD1 suppresses breast cancer progression by attenuating Wnt/β-catenin signaling. (A, B) Representative images (A) and quantification (B) of IHC staining for β-catenin in xenograft tumors. One-sided unpaired t-test. (C) Immunoblot analysis of cytoplasmic and nuclear fractions showing increased nuclear β-catenin upon FXYD1 knockdown. (D) Immunoblot analysis of β-catenin, CD44, c-Myc and Met in FXYD1 knockdown cells. (E, F) qRT-PCR analysis of indicated genes in FXYD1-overexpressing (E) and FXYD1-silenced (F) cells. One-sided unpaired t-test. (G) Immunoblot analysis of EMT markers in cells transduced with vector or FXYD1, and in FXYD1-overexpressing cells further transduced with β-catenin. E-cadherin is not detectable in MDA-MB-231 cells (see legend of Figure 2H). (H) qRT-PCR analysis

of indicated genes in MDA-MB-231 and BT-549 cells transduced with vector or FXYD1, and in FXYD1-overexpressing cells further transduced with β-catenin. One-way ANOVA. Data are presented as the mean ± SD (n = 3 independent experiments). ***p* < 0.01, ****p* < 0.001.

Figure S7

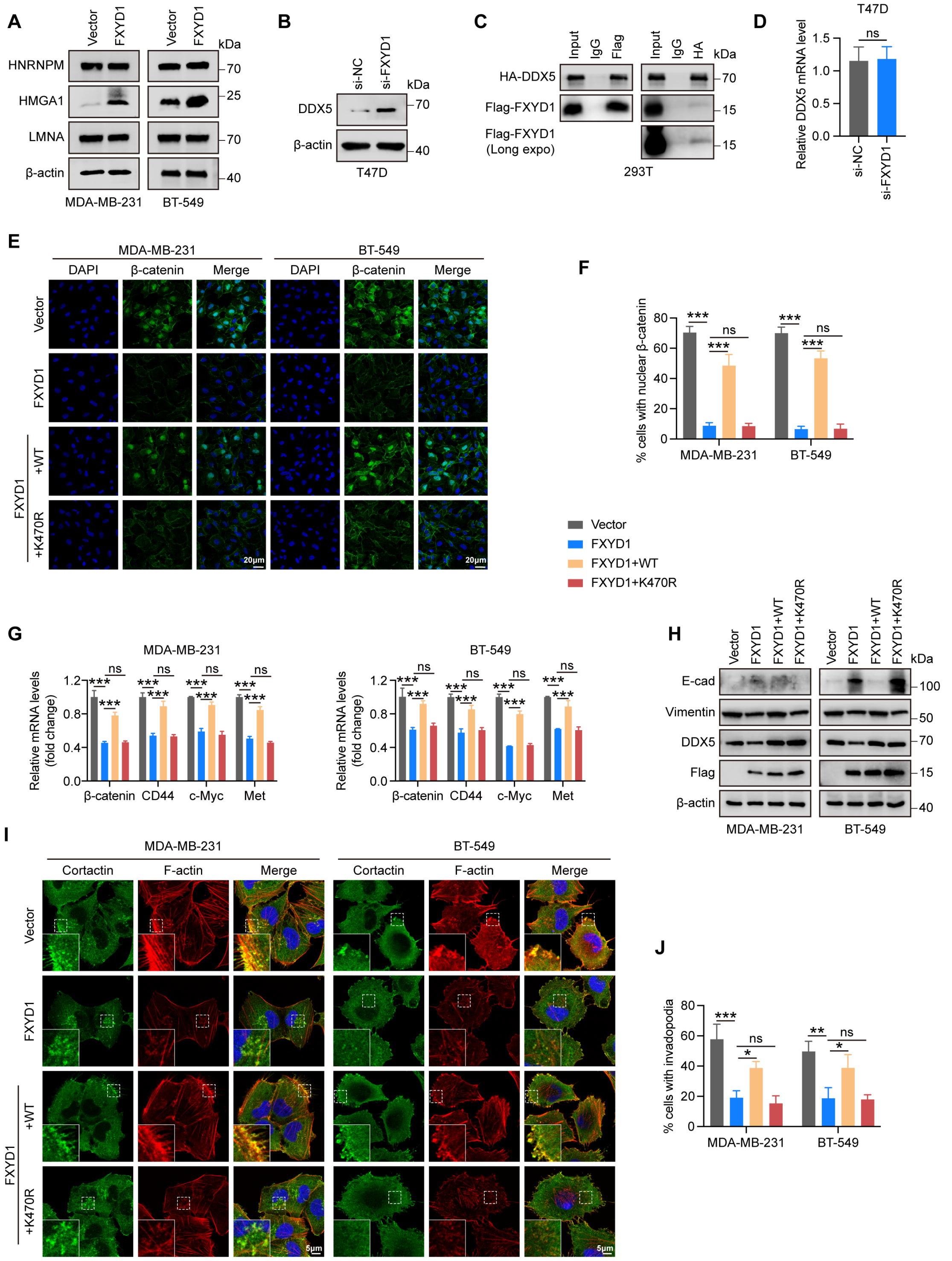

Supplementary Figure 7. FXYD1 suppresses Wnt/β-catenin signaling by downregulating DDX5. (A) Immunoblot analysis of HNRNPM, HMGA1 and LMNA in MDA-MB-231 and BT-549 cells transduced with vector or FXYD1. (B) Immunoblot analysis of DDX5 in FXYD1 knockdown cells using siRNA targeting FXYD1. (C) Co-IP assays detecting the interaction between ectopically expressed Flag-FXYD1 and HA-DDX5 in HEK293T cells. (D) qRT-PCR analysis of DDX5 in FXYD1 knockdown cells using siRNA targeting FXYD1. One-sided unpaired t-test. (E, F) IF staining (E) and quantification (F) for nuclear β-catenin in MDA-MB-231 and BT-549 cells transduced with Vector or FXYD1, as well as FXYD1-overexpressing cells further transduced with DDX5-WT or K470R. One-way ANOVA. (G) qRT-PCR analysis of indicated genes in the indicated groups. One-way ANOVA. (H) Immunoblot analysis of EMT markers in the indicated groups. E-cadherin is not detectable in MDA-MB-231 cells (see legend of Figure 2H). (I, J) IF analysis (I) and quantification of invadopodia formation (J) in the indicated groups. One-way ANOVA. Data are presented as the mean ± SD (n = 3 independent experiments). ns, not significant, *$p < 0.05$, **$p < 0.01$, ***$p < 0.001$.

Figure S8

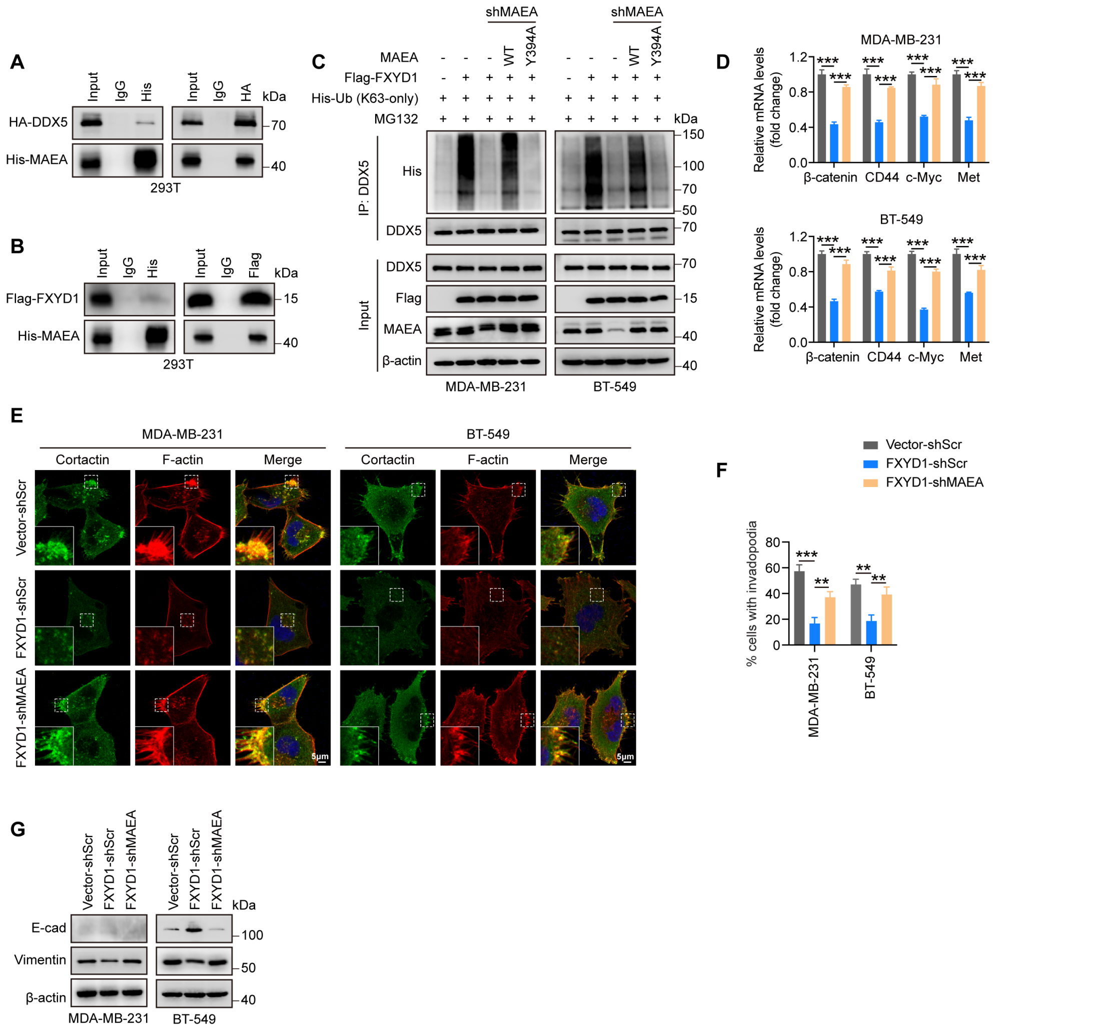

Supplementary Figure 8. FXYD1 regulates DDX5-β-catenin axis through MAEA-dependent ubiquitination. (A) Co-IP assays detecting the interaction between ectopically expressed His-MAEA and HA-DDX5 in HEK293T cells. (B) Co-IP assays detecting the interaction between ectopically expressed His-MAEA and Flag-FXYD1 in HEK293T cells. (C) MDA-MB-231 and BT-549 cells with MAEA knockdown were reconstituted with MAEA-WT or MAEA-Y394A, and co-expressed with Flag-FXYD1 and His-tagged K63-only ubiquitin, followed by MG132 treatment (20 μM, 8 h). DDX5 was immunoprecipitated, and ubiquitination was assessed by immunoblotting with the indicated antibodies. (D) qRT-PCR analysis of indicated genes in vector or FXYD1-overexpressing cells transduced with scrambled or MAEA shRNA. (E–G) FXYD1-overexpressing cells or vector controls were transduced with MAEA shRNA or

scrambled shRNA. Invadopodia formation and EMT progression were analyzed by IF (E, F) and immunoblotting (G). E-cadherin is not detectable in MDA-MB-231 cells (see legend of Figure 2H). Data are presented as the mean ± SD (n = 3 independent experiments). ns, not significant, **$p < 0.01$, ***$p < 0.001$ vs. indicated controls in one-way ANOVA.

Figure S9

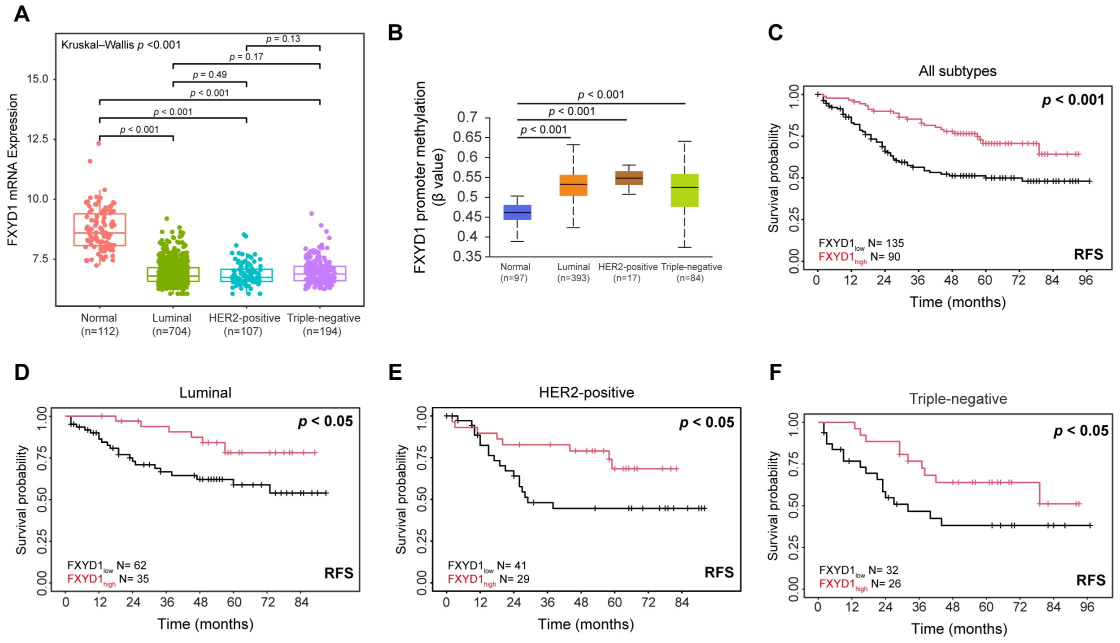

Supplementary Figure 9. Subtype-stratified expression, promoter methylation, and prognosis of FXYD1 in breast cancer. (A) FXYD1 mRNA expression across molecular subtypes and normal breast tissues in TCGA cohort. (B) FXYD1 promoter methylation levels across breast cancer subtypes and normal tissues in TCGA cohort. (C–F) Kaplan–Meier analysis showing that FXYD1 expression levels correlate with improved RFS across molecular subtypes in our clinical cohort.

Supplementary Tables

Supplementary Table 1: Univariate analysis for factors associated with RFS.

Characteristics	Univariate	
	HR (95% CI)	*p*-Value
Age years (⩾50 versus <50)	0.941 (0.608–1.455)	0.784
Menopause (Yes versus No)	1.126 (0.728–1.743)	0.594
Histological grade (III versus I-II)	1.412 (0.885–2.252)	0.147
Tumor size (T3 versus T1-2)	1.339 (0.806–2.223)	0.260
Nodal status (Positive versus Negative)	3.129 (1.809–5.412)	<0.001
Histological Type (Others versus Ductal/NST)	1.620 (0.654–4.009)	0.297
ER (Positive versus Negative)	0.678 (0.429–1.072)	0.097
PR (Positive versus Negative)	0.521 (0.308–0.880)	0.015
HER2 status (Positive versus Negative)	1.134 (0.732–1.756)	0.573
Ki67 expression % (⩾14 versus <14)	1.273 (0.774–2.093)	0.341
FXYD1 (High versus Low)	0.436 (0.270–0.703)	<0.001

Supplementary Table 2: Multivariate analysis for factors associated with RFS.

Characteristics	Multivariate	
	HR (95% CI)	*p*-Value
Tumor size (T3 versus T1-2)	1.462 (0.863–2.478)	0.158
Nodal status (Positive versus Negative)	3.244 (1.856–5.670)	<0.001
ER (Positive versus Negative)	0.984 (0.509–1.904)	0.962
PR (Positive versus Negative)	0.419 (0.194–0.903)	0.026
Ki67 expression % (⩾14 versus <14)	1.062 (0.630–1.791)	0.820
FXYD1 (High versus Low)	0.420 (0.257–0.687)	<0.001

Supplementary Table 3: Clinicopathological characteristics of the paired specimens used for BSP analysis (n = 3 pairs).

Case	Age (years)	Histological type	Histological Grade	ER	PR	HER2	Molecular subtype
Patient #1	65	Ductal/NST	3	+	+	-	Luminal
Patient #2	63	Ductal/NST	3	-	-	+	HER2-positive
Patient #3	62	Ductal/NST	3	-	-	-	TNBC

Supplementary Table 4: Cell sources and Research Resource Identifiers.

Cell line	Source	Research Resource Identifier
MDA-MB-231	ATCC HTB-26	CVCL_0062
BT-549	ATCC HTB-122	CVCL_1092

T47D	ATCC HTB-133	CVCL_0553
SK-BR-3	ATCC HTB-30	CVCL_0033
BT-474	ATCC HTB-20	CVCL_0179
MDA-MB-468	ATCC HTB-132	CVCL_0419
MCF-7	ATCC HTB-22	CVCL_0031
MCF-10A	ATCC CRL-10317	CVCL_0598
HEK293T	ATCC CRL-3216	CVCL_0063

Supplementary Table 5: List of antibodies with their sources and experimental conditions.

Primary antibody	Source	Application	Catalogue number	Dilution	Company
FXYD1	Rabbit	WB	ab191397	1:2000	Abcam
FXYD1	Rabbit	IHC	ab191397	1:50	Abcam
FXYD1	Rabbit	IF	ab191397	1:200	Abcam
FXYD1	Rabbit	IP	ab191397	1:100	Abcam
β-actin	Mouse	WB	sc-47778	1:2000	Santa Cruz
E-cadherin	Mouse	WB	20874-1-AP	1:1000	Proteintech
E-cadherin	Mouse	IHC	20874-1-AP	1:200	Proteintech
N-cadherin	Mouse	WB	22018-1-AP	1:1000	Proteintech
N-cadherin	Mouse	IHC	22018-1-AP	1:200	Proteintech
Vimentin	Mouse	WB	sc-32322	1:1000	Santa Cruz
Snail	Rabbit	WB	3879	1:1000	Cell signaling technology
Ki-67	Rabbit	IHC	27309-1-AP	1:2000	Proteintech
β-catenin	Rabbit	WB	8480	1:1000	Cell signaling technology
β-catenin	Rabbit	IHC	8480	1:50	Cell signaling technology
β-catenin	Rabbit	IF	8480	1:100	Cell signaling technology
c-Myc	Rabbit	WB	13987	1:1000	Cell signaling technology
CD44	Mouse	WB	3570	1:1000	Cell signaling technology
HMGA1	Rabbit	WB	ab129153	1:1000	Abcam
HNRNPM	Rabbit	WB	88145	1:1000	Cell signaling technology
LMNA	Mouse	WB	4777	1:1000	Cell signaling technology
Met	Rabbit	WB	8198	1:1000	Cell signaling technology
DDX5	Rabbit	WB	9877	1:1000	Cell signaling technology
DDX5	Rabbit	IHC	9877	1:200	Cell signaling technology
DDX5	Rabbit	IF	9877	1:200	Cell signaling technology
DDX5	Rabbit	IP	9877	1:100	Cell signaling technology
MAEA	Rabbit	WB	28363-1-AP	1:1000	Proteintech
MAEA	Rabbit	IF	28363-1-AP	1:200	Proteintech
MAEA	Rabbit	IP	28363-1-AP	1:100	Proteintech
Ubiquitin	Rabbit	WB	3936	1:1000	Cell signaling technology
Cortactin	Rabbit	IF	11381-1-AP	1:200	Proteintech
HA	Rabbit	WB	51064-2-AP	1:5000	Proteintech

HA	Rabbit	IP	51064-2-AP	1:100	Proteintech
Flag	Mouse	WB	66008-4-Ig	1:5000	Proteintech
Flag	Mouse	IP	66008-4-Ig	1:100	Proteintech
His	Mouse	WB	66005-1-Ig	1:5000	Proteintech
His	Mouse	IP	66005-1-Ig	1:100	Proteintech

Supplementary Table 6: List of primers used for qRT-PCR.

Primer	Sequence
Human FXYD1 Forward	5’-GCGTCTCTTGGCCACATCTT-3’
Human FXYD1 Reverse	5’-CTTCATCGGGTTCCCCAGTC-3’
Human DDX5 Forward	5’-CTGGACCCTACCTACATCCTG-3’
Human DDX5 Reverse	5’-GGCATCCAAAAAGCCACGG-3’
Human β-catenin Forward	5’-GTTCAGTTGCTTGTTCGTGC-3’
Human β-catenin Reverse	5’-GTTGTGAACATCCCGAGCTAG-3’
Human CD44 Forward	5’-CTGCCGCTTTGCAGGTGTA-3’
Human CD44 Reverse	5’-CATTGTGGGCAAGGTGCTATT-3’
Human c-Myc Forward	5’-GGACCCGCTTCTCTGAAAG-3’
Human c-Myc Reverse	5’-GTCGAGGTCATAGTTCCTGTTG-3’
Human Met Forward	5’-AGAAGACTCCTACAACCCGAATACT-3’
Human Met Reverse	5’-TCCACCTCATCATCAGCGTTATC-3’
Human β-actin Forward	5’-GTCTTCCCCTCCATCGTG-3’
Human β-actin Reverse	5’-AGGGTGAGGATGCCTCTCTT-3’
